# A Novel Approach to Map the Causal Impact of Brain Stimulation on Semantic Processing With Language Models

**DOI:** 10.1162/NOL.a.244

**Published:** 2026-05-05

**Authors:** Andrea Bruera, Gesa Hartwigsen

**Affiliations:** Research Group Cognition and Plasticity, Max Planck Institute for Human Cognitive and Brain Sciences, Leipzig, Germany; Cognitive and Biological Psychology, Wilhelm Wundt Institute for Psychology, Leipzig University, Leipzig, Germany

**Keywords:** brain stimulation, causality, language models, semantics, semantic models, semantic similarity, surprisal, transcranial magnetic stimulation (TMS)

## Abstract

Noninvasive brain stimulation studies on semantic cognition hold the promise of revealing the functional relevance of brain areas through causal intervention. A primary challenge, however, is that findings are often interpreted through binary distinctions between sets of stimuli (e.g., related/unrelated words, same/different semantic category). This approach ignores the analysis of individual words, which mirrors every day language use and is crucial for understanding semantic cognition. In this work, we used semantic similarity, as measured by a language model, to investigate how transcranial magnetic stimulation (TMS) effects on semantic cognition unfold at the level of individual words. We reanalyzed five publicly available TMS data sets, covering multiple stimulation sites and lexical semantics tasks. We propose a simple methodology that can straightforwardly be applied to any TMS experiment on semantic cognition and showcase its potential to generate new insights. We modeled trial-level response times using the language model and computed the correlation between the two. We also repeated the analyses for two lower-level variables (word frequency and length). Importantly, for each data set, we compared correlations for effective and control (sham or vertex) stimulation conditions. We found that, for the language model, correlation was almost always significantly different depending on the type of stimulation (effective or control). Our results provide evidence that the stimulation effect interacts with the meaning of individual words. However, a similar pattern emerged in some cases for word frequency and length, suggesting that the effects of TMS on cognition can be widespread, well beyond their intended functional target. Collectively, our results demonstrate that language models provide new insight into the impact of neurostimulation on semantic processing, complementing standard measures.

## INTRODUCTION

Semantic cognition is central to human communication and interaction in everyday life. The meaning of the words we use can be captured by dividing them into distinct categories: animate as opposed to inanimate; objects as opposed to actions; concrete as opposed to abstract words. Such binary distinctions among sets of words have proven to be an insightful window into the way our brain processes semantics (Binder et al., 2005; Caramazza & Shelton, 1998; Martin, 2007). Yet, words are represented and used in ways that go well beyond dichotomies and categories (Huth et al., 2012; Trott & Bergen, 2023; van Hoef et al., 2023). For instance, a cat and an elephant belong to the same category—animals. However, while I regularly interact with cats, I have never seen an elephant. The vast differences in real-life sensory and social experiences I have with the two dramatically change the way my brain stores and accesses the meaning of the word “cat” as opposed to “elephant” (Brysbaert et al., 2017; Charest et al., 2014; Renoult et al., 2019; Yonelinas, 2002). This impact is so pronounced that the fact that they belong, ontologically, to the same category may become almost irrelevant (Bozeat et al., 2002; Bruera & Poesio, 2025; Hoffman, 2018; Rogers et al., 2015).

In recent years, the cognitive neuroscience of language has started taking seriously the investigation of how our brains store and use individual concepts, as opposed to binary, mutually exclusive categories (Fernandino et al., 2022; Huth et al., 2016; Mitchell et al., 2008; Pereira et al., 2018). Despite decades of effort, it is a longstanding, unresolved question how semantic knowledge—which has been shown to involve disparate pieces of information (e.g., sensory, motor, social, abstract) across distant areas of the brain—is retrieved, brought together and used in natural language and behavior (Binder et al., 2009; Calzavarini, 2024; Lambon Ralph et al., 2017). This puzzle still eludes our understanding, among other reasons, because it has been extremely hard to obtain consistent and reliable evidence about the causal role of specific areas: which brain area is playing a necessary role and when (Drijvers et al., 2025; Jackson, 2021; Schroën et al., 2023).

To address the issue of causality, noninvasive brain stimulation (NIBS) is a key technique in the cognitive neuroscientist’s toolbox (Bergmann et al., 2016). In particular, transcranial magnetic stimulation (TMS) has been used for more than two decades now to transiently modulate brain function and probe the relevance of specific brain areas for distinct cognitive operations (Bergmann & Hartwigsen, 2021; Pitcher et al., 2021). In a typical cognitive experiment, healthy participants repeat the same task with effective and ineffective stimulation of a given area. This allows the researcher to probe the question of what would be the effect on a subject’s behavior during a cognitive task if the regular functioning brain area *X* was altered. Relative to the study of patients with brain lesions, TMS-induced perturbations allow for relatively precisely localized stimulation effects, targeting individual brain regions and allowing to formulate very specific research questions about the relevance of specific brain areas for different cognitive subprocesses at different time points (Hallett, 2007; Hartwigsen, 2015; Jefferies, 2013; Valero-Cabré et al., 2017).

To address theoretical questions in semantics, TMS has been used in a variety of ways. First, to complement results in stroke patients, either trying to induce a category-specific deficit (Cattaneo et al., 2010; Pobric et al., 2010; Thompson et al., 2017) or to solve conflicting reports (Devlin et al., 2003; Finocchiaro et al., 2015). Second, to test existing models of semantics in the brain—such as the so-called hub-and-spoke and the controlled semantic cognition theories of semantic processing (Chiou et al., 2018; Jefferies, 2013) or the embodied cognition framework (Pulvermüller et al., 2005). Third, framing TMS perturbation in a network-based approach to assess its effects beyond the stimulated brain region (Binney & Lambon Ralph, 2015; Hartwigsen et al., 2016). Finally, to understand the exact role in semantic processing of specific areas (e.g., Davey et al., 2015; Gatti et al., 2020; Pobric et al., 2008).

However, the impact of TMS studies on semantics is significantly limited by the focus on the comparisons between dichotomous categories or conditions (such as semantically related/unrelated, same/different semantic category), missing on the uniqueness of the meaning of individual words. Reaching this level of concept-specific granularity in our understanding of the causal promise of TMS bears exciting potentials: not only revealing previously unattainable evidence about semantic cognition, but also bringing this stimulation approach closer to everyday language use.

**Figure 1.**
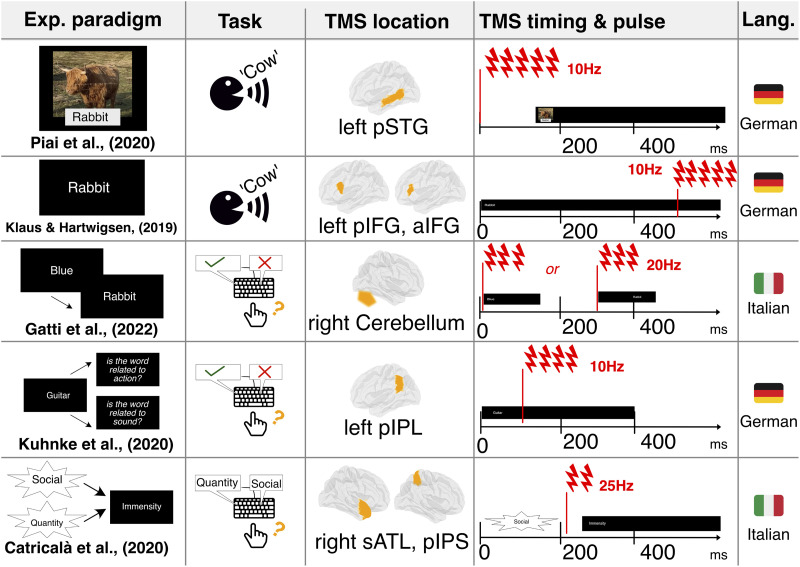
Main details of the used data sets.

In this work, we reanalyzed five existing TMS data sets on semantic cognition to reveal how stimulation affected the processing of individual words (i.e., words in isolation, without any larger linguistic context). To ensure reliability of our results, we gathered as many publicly available data sets as possible. We could cover two languages (Italian and German), seven brain areas associated with semantic processing, and five different semantic tasks, involving both comprehension and production tasks. The main characteristics of each data set can be visualized in Figure 1. As a measure of cognitive processing related to processing of individual words, we used response times (RTs), which is the most commonly used index of cognitive effort in TMS studies (Devlin et al., 2003; Jefferies, 2013; Papeo et al., 2013; Qu et al., 2022).

To quantify the effects of individual word meaning on each trial, we adopted an information-based framework—which is commonplace in functional neuroimaging and electrophysiological approaches (Kriegeskorte & Bandettini, 2007; Naselaris et al., 2011) but not in TMS studies, at least for language (Romei et al., 2016). We measured the correlation between cognitive effort and different types of information (semantic, frequency, and orthographic information) and probed whether this correlation was affected by TMS. We also used a simple procedure (hierarchical bootstrapping with subsampling; Saravanan et al., 2020) to provide standardized measurements of correlation across studies and conditions to even out differences in sample sizes that could bias the summary statistics (Minarik et al., 2016; Poldrack et al., 2017). The key advantages of this approach are twofold.

First, since it is based on correlation, it allows us to use continuous measurements for each type of information, thus recognizing and accounting for the specificity and uniqueness of the individual words in each trial (Bruffaerts et al., 2019; Gao et al., 2023).

Second, it operates at a more abstract level than simple RTs, which is by contrast commonplace in TMS research (Hartwigsen, 2015; Qu et al., 2022). A difference in RTs across conditions between effective and control stimulation is traditionally considered to be—in the context of a TMS study on semantic cognition—a measure of a semantic effect. However, such differences could, in principle, be explained by different factors (i.e., distinct types of information). By formulating explicit, competing models of cognitive processing and evaluating how their fit with cognitive effort is affected by neurostimulation, an information-based approach enables to directly test whether the TMS effect on cognitive effort can be attributed to modulation of semantic information processing (Kriegeskorte & Douglas, 2018; Lopopolo et al., 2024; Naselaris & Kay, 2015; Peelen & Downing, 2023).

Importantly, this also allows to neutralize the effect of specific experimental parameters such as the stimulation timing, or the frequency and the number of pulses, which are known to have low-level effects on behavioral measures like RTs (Beynel et al., 2019; Hartwigsen & Silvanto, 2023; Pell et al., 2011). As pointed out in Beynel et al. (2019), the concept of brain stimulation is appealingly simple; in fact, its experimental setup is extremely complex and variable, with a large number of possible stimulation parameters that are likely to interact and influence each other. Some rules of thumb are often applied (e.g., frequency ≤1 Hz for a decrease in cortical excitability and ≥5 Hz for an increase), but they are not grounded in solid evidence outside the motor cortex and they often lead to the opposite effect in studies of cognition (see Hartwigsen & Silvanto, 2023). Thus, in practice, such differences in the actual implementation of brain stimulation in each experiment (i.e., the set of parameters used) reduce the possibility to compare results directly, because the stimulation-induced effects on the brain vary widely across experiments. What is needed to compare multiple studies is a unifying, higher-level hypothesis about the cognitive (i.e., not neural) process that is being affected.

To address this issue, we chose similarity as an explicit, higher-level general model of the cognitive processes involved in linguistic and semantic cognition. Similarity is not only a fundamental modeling concept in cognitive neuroscience (Edelman, 1998; Tversky, 1977) but also extremely parsimonious: It can be applied to all levels of processing and is therefore perfectly suited to a complex cognitive phenomenon such as language.

For semantic information, we modeled the cognitive effort induced by each trial with a language model or a distributional semantic model (Boleda, 2020; Connell & Lynott, 2024; Lenci, 2018)—belonging to a family of computational models that have been shown to excel at capturing cognitive semantic processing (Goldstein et al., 2024; Huth et al., 2016; Lenci et al., 2022; Schrimpf et al., 2021; Tuckute et al., 2024). For each trial, we made the simple assumption that the cognitive effort should be related to the semantic similarity between the concepts involved in it—an expectation justified by a large body of previous work (Bhatia & Aka, 2022; Wingfield & Connell, 2022). We also implemented in the same way the two control models, capturing lower levels of processing: one using word frequency (i.e., statistical information), and the other using word length (i.e., orthography).

**Figure 2.**
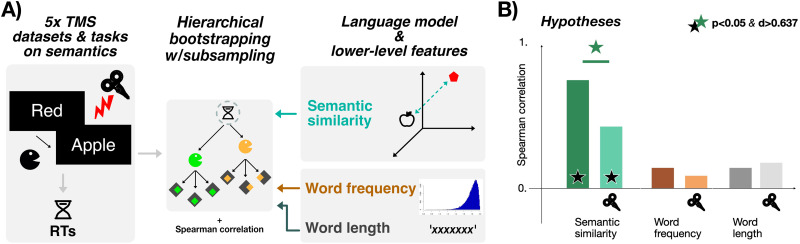
Methodology used to measure brain stimulation effects on semantic cognition (A) and experimental hypotheses that we expected to hold across studies (B).

Then, separately for each study, model, and stimulation condition, we computed the correlation between RTs and each model (language model, word frequency, word length). Finally, we tested whether the effect of TMS on the fit between RTs and each type of information was significant: If that were the case, we interpreted it as indicating that processing of that information was affected by stimulation. For a simplified visualization of our approach, see Figure 2A.

Inspired by previous neurostimulation studies (e.g., Gatti et al., 2022), we tested two hypotheses (see Figure 2B). First, the semantic similarity model should show above-chance correlation with cognitive effort for all tasks, in the condition where no effective stimulation was applied—indicating that individual word-level semantics was effectively captured in regular, unaltered cognition; regarding word frequency and length, we had no specific expectation. Second, TMS should induce a significant modulation of the fit only for the semantic similarity model, indicating that the TMS effect was specific to semantics—and that the directionality of this effect should be a reduction in correlation after stimulation (see Gatti et al., 2022).

## RESULTS

For simplicity, we divided the presentation of the results in four sections: first, reporting the two data sets on language production (Klaus & Hartwigsen, 2019; Piai et al., 2020); then, the two data sets on semantic judgments without priming (Gatti et al., 2022; Kuhnke et al., 2020); and finally, the semantic priming study (Catricalà et al., 2020). We added an additional set of analyses since, contrary to our expectations, the effect of TMS turned out to be significant also on nonsemantic variables for some data sets. Therefore, we repeated the analyses after removing the variance that could be explained by them (both word length and word frequency) from the RTs. This allowed us to measure the specific effect of TMS on semantics.

We report the full set of statistics in the main text (Spearman correlation, *p* value, effect size, 95% confidence intervals (CIs)) only if two conditions were fulfilled: a *p* value smaller than 0.05 and an effect size larger than *d* > 0.632, which corresponds to the 25th percentile value estimated from significant results across the cognitive neuroscience literature—which we interpret as indicating a small effect. We report the full set of statistics in the Supplementary Materials (Supporting Information can be found at https://doi.org/10.1162/NOL.a.244; additional Results section, Tables C15–C19).

Note that if the correlation between a model and the RTs is not statistically significant and the data do not show at least a small effect in the control condition (sham/vertex), the comparisons between conditions are not interpretable because of the lack of reliability of the model’s ability to explain the behavioral data. In such cases, we do not report statistics in the main text.

Also, while in the text for simplicity we refer to semantic (dis)similarity, word frequency, and word length, note that the actual implementation of the models’ measures was adapted so that the expected correlation with cognitive effort (indexed by RTs) would be positive: Therefore, in practice, we computed the correlation between RTs and semantic dissimilarity (1 − similarity; the less similar two words, the larger the cognitive effort), RTs and negative word frequency (the less frequent a word, the larger the cognitive effort), and RTs and word length.

### TMS Increases Sensitivity to Semantic (Dis)similarity in Semantic Production Tasks

**Figure 3.**
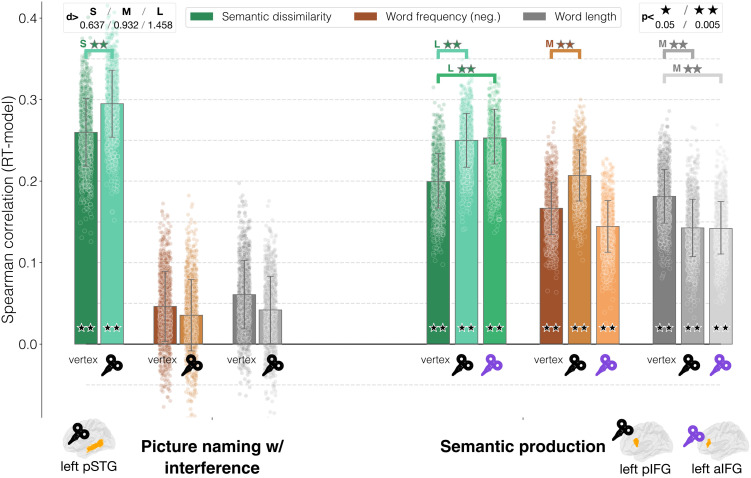
Semantic production tasks—main results. The bar heights correspond to the average Spearman correlation between each model and the response times across all bootstrap iterations; error bars represent the standard deviation; the dots correspond to the Spearman correlation for each individual iteration.

We start from the picture–word interference task of Piai et al. (2020) (Figure 3, left). Semantic dissimilarity correlated significantly with RTs for both vertex (*ρ* = 0.257, *p* = 0.004, *d* = 6.16, 95% CIs [0.255, 0.260]) and left posterior superior temporal gyrus (pSTG) (*ρ* = 0.293, *p* = 0.004, *d* = 7.12, 95% CIs [0.290, 0.296]), while this was not the case for word frequency (vertex: *ρ* = 0.0459, left pSTG: *ρ* = 0.0331) nor word length (vertex: *ρ* = 0.0588, left pSTG: *ρ* = 0.0384). Similarly, the effect of TMS was significant and showed at least a small effect only for semantic dissimilarity (*d* = −0.85, 95% CIs [−0.93, −0.78], *p* = 0.003), while it had a negligible effect for word frequency, however, with the opposite direction as semantic similarity (*d* = 0.288, *p* = 0.003). The same happened with word length (*d* = 0.471, *p* = 0.003). Contrary to our hypothesis, effective TMS increased the correlation between semantic dissimilarity and RTs—suggesting that after stimulation, the semantic (dis)similarity between the trial-relevant items plays a more important role in determining the amount of cognitive effort involved in solving the task.

We found different results for the semantic production task (Klaus & Hartwigsen, 2019; Figure 3, right). Here, all models—both semantic and nonsemantic—were significantly correlated with RTs, in all conditions. The highest correlation value in the control condition was found for semantic dissimilarity (vertex: *ρ* = 0.199, *p* = 0.0043, *d* = 6.08, 95% CIs [0.197, −0.201]; posterior inferior frontal gyrus (pIFG): *ρ* = 0.254, *p* = 0.0043, *d* = 7.59, 95% CIs [0.251, 0.256]; anterior inferior frontal gyrus (aIFG): *ρ* = 0.258, *p* = 0.0043, *d* = 7.76, 95% CIs [0.256, 0.26]). The second highest correlation for vertex was found for word length (vertex: *ρ* = 0.181, *p* = 0.0043, *d* = 5.56, 95% CIs [0.179, 0.183]; pIFG: *ρ* = 0.143, *p* = 0.0043, *d* = 4.07, 95% CIs [0.141, 0.146]; aIFG: *ρ* = 0.142, *p* = 0.0043, *d* = 4.36, 95% CIs [0.14, 0.144]), followed by word frequency (vertex: *ρ* = 0.165, *p* = 0.0043, *d* = 5.06, 95% CIs [0.163, 0.167]; pIFG: *ρ* = 0.205, *p* = 0.0043, *d* = 6.2, 95% CIs [0.203, 0.207]; aIFG: *ρ* = 0.146, *p* = 0.0043, *d* = 4.63, 95% CIs [0.144, 0.148]). When looking at differences among conditions, all comparisons were statistically significant, showing at least a medium effect. However, the directionality of the effects varied noticeably, showing an interaction between stimulation and type of information being processed: For semantic dissimilarity, TMS consistently increased correlation between RTs and semantic similarity, coherently with the results reported above on picture–word interference (pIFG: *d* = −1.647, 95% CIs [−1.74, −1.55], *p* = 0.0032; aIFG: *d* = −1.777, 95% CIs [−1.88, −1.68], *p* = 0.0032). For word length, TMS had the opposite effect, always decreasing correlations—thus indicating that the same stimulation can affect the processing of different pieces of information in different ways (pIFG: *d* = 1.108, 95% CIs [1.03,1.19], *p* = 0.0032; aIFG: *d* = 1.186, 95% CIs [1.11, 1.27], *p* = 0.0032). This was even more strongly evident for word frequency, where the effect diverged depending on the target area: for pIFG TMS only, correlation with word frequency increased (pIFG: *d* = −1.22, 95% CIs [−1.31, −1.15], *p* = 0.0032), while results went in the other direction and were only close to having a small effect for the aIFG (aIFG: *d* = 0.574, 95% CIs [0.51, 0.64], *p* = 0.01).

Overall, the results obtained for language production provided clear evidence for a significant effect on semantics. However, they contradicted our hypothesis that semantic (dis)similarity should be decreased as TMS should induce noise in the system. Rather, our results show that TMS increased the amount of variance explained by this value and this effect. Also, this was not specifically affecting semantic processing, but also lower-level processes (recognition, as captured by word frequency, and orthography, via word length).

### Semantic Judgments Show That TMS and Task Interact

**Figure 4.**
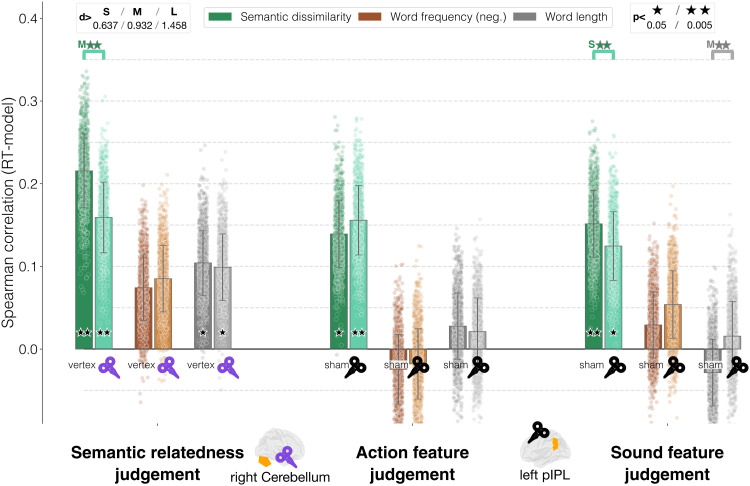
Semantic judgment tasks—main results. The bar heights correspond to the average Spearman correlation between each model and the response times across all bootstrap iterations; error bars represent the standard deviation; the dots correspond to the Spearman correlation for each individual iteration.

Results for semantic judgment tasks are shown in Figure 4. On the left side, we report correlations for the relatedness judgment task of Gatti et al. (2022). We confirmed the original study’s results, which also motivated some of our hypotheses. We found that semantic dissimilarity was significantly correlated with RTs (vertex: *ρ* = 0.214, *p* = 0.0044, *d* = 4.76, 95% CIs [0.211, 0.216]; right cerebellum: *ρ* = 0.159, *p* = 0.0044, *d* = 3.65, 95% CIs [0.157, 0.162]). Moreover, TMS significantly affected semantic processing by reducing the correlation between semantics and RTs after stimulation (*d* = 1.184, 95% CIs [1.1, 1.27], *p* = 0.003). Word length was significantly correlated with RTs in both conditions (vertex: *ρ* = 0.107, *p* = 0.0314, *d* = 2.58, 95% CIs [0.104, 0.11]; right cerebellum: *ρ* = 0.0975, *p* = 0.042, *d* = 2.373, 95% CIs [0.094, 0.1]); however, the effect of TMS on processing of orthographic information was negligible (*d* = 0.43, 95% CIs [0.37, 0.5], *p* = 0.0032). Finally, correlation between word frequency and RTs was never statistically significant (vertex: *ρ* = 0.0745; right cerebellum: *ρ* = 0.0838). In this respect, the results from this data set fit our hypotheses: Semantic (dis)similarity explained RTs during the task significantly better than chance, and such semantic information was selectively targeted by stimulation.

A similar picture emerged for the sound and action judgments data set of Kuhnke et al. (2020), with the added value of showing a critical interaction between stimulation and task. More specifically, for the action judgment task (Figure 4, middle), only semantic dissimilarity was significantly correlated with RTs (sham: *ρ* = 0.138, *p* = 0.0044, *d* = 3.207, 95% CIs [0.136, 0.141]; left posterior inferior parietal lobe (pIPL): *ρ* = 0.153, *p* = 0.0044, *d* = 3.706, 95% CIs [0.151, 0.156]), and neither of the lower-level features did so (word frequency: sham, *ρ* = −0.024; left pIPL, *ρ* = −0.0169; word length: sham, *ρ* = 0.029; left pIPL, *ρ* = 0.021). However the effect of TMS on semantics during the action task, as measured by the effect size, was negligible (*d* = −0.353, *p* = 0.003).

**Figure 5.**
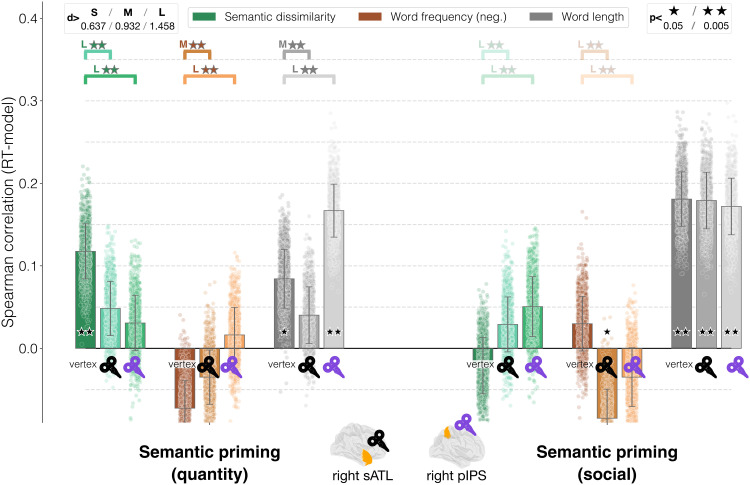
Semantic priming tasks—main results. The bar heights correspond to the average Spearman correlation between each model and the response times across all bootstrap iterations; error bars represent the standard deviation; the dots correspond to the Spearman correlation for each individual iteration.

In contrast, for the sound task, the results matched the expected effects (Figure 5, right side): Semantic dissimilarity was not only significantly correlated with RTs (sham: *ρ* = 0.15, *p* = 0.0044, *d* = 3.69, 95% CIs [0.148, 0.153]; left pIPL: *ρ* = 0.123, *p* = 0.0044, *d* = 2.96, 95% CIs [0.121, 0.126]) but was also significantly affected by TMS, causing the expected decrease in correlation after stimulation (*d* = 0.644, 95% CIs [0.58, 0.71], *p* = 0.003). Neither word frequency nor word length show significant correlations in the sham condition (word frequency: *ρ* = 0.028; word length: *ρ* = −0.0282).

### Language Models Capture TMS Effects on Semantic Priming for Quantity Words

Finally, the results for the priming experiment are reported in Figure 5. Starting from semantic priming on quantity concepts (Figure 5, left side), semantic dissimilarity emerged as the closest match to RTs (vertex: *ρ* = 0.117, *p* = 0.0044, *d* = 3.36, 95% CIs [0.115, 0.12]), followed by word length (vertex: *ρ* = 0.0837, *p* = 0.024, *d* = 2.46, 95% CIs [0.0816, 0.085]). Word frequency, on the contrary, did not correlate significantly with RTs (vertex: *ρ* = −0.0732). Stimulation had a strong effect on semantic processing, both for the right posterior intraparietal sulcus (pIPS) and the right superior anterior temporal lobe (sATL). In both cases, TMS decreased the match between semantic dissimilarity and RTs (right pIPS: *d* = 2.54, *p* = 0.0032, 95% CIs [2.42, 2.68]; right sATL: *d* = 2.073, *p* = 0.0032, 95% CIs [1.96, 2.18]). This is consistent with the results reported in Figure 4—in all these data sets, the experimental task required subjects to provide semantic judgments. For word length, again consistently with the results of Figure 4, the effect of TMS depended on the target: When stimulating the right sATL, the correlation was significantly decreased, while for the right pIPS, orthographic information was significantly correlated with RTs (right pIPS: *d* = −2.419, *p* = 0.0032, 95% CIs [−2.54, −2.3]; right sATL: *d* = 1.41, *p* = 0.0032, 95% CIs [1.33, 1.5]). This aligned only partially with our hypotheses: The TMS effect on semantics matched our expectations and revealed that not only the right pIPS, but also the right sATL, played a role in semantic processing of quantity words. However, in contrast to our expectations, orthographic information was also significantly affected by TMS, although in partially different directions.

For social concepts (Figure 5, right side), our analyses revealed a different picture: Variation in RTs seemed to be largely due to word length, the only model that was significantly correlated with RTs (vertex: *ρ* = 0.181, *p* = 0.0044, *d* = 5.52, 95% CIs [0.179, 0.183]; right pIPS: *ρ* = 0.172, *p* = 0.0044, *d* = 5.17, 95% CIs [0.17, 0.174]; right sATL: *ρ* = 0.178, *p* = 0.0044, *d* = 5.3, 95% CIs [0.176, 0.18]). However, TMS did not seem to have an effect on orthographic processing either (right pIPS: *d* = 0.275, *p* = 0.063, 95% CIs [0.21, 0.34]; right sATL: *d* = 0.0911, *p* = 0.336, 95% CIs [0.03, 0.15]); we interpret this as suggesting that none of the models used was, in fact, able to capture the type of processing affected by TMS.

### TMS Effects on Semantic Processing Are Still Present After Accounting for Word Length and Frequency

**Figure 6.**
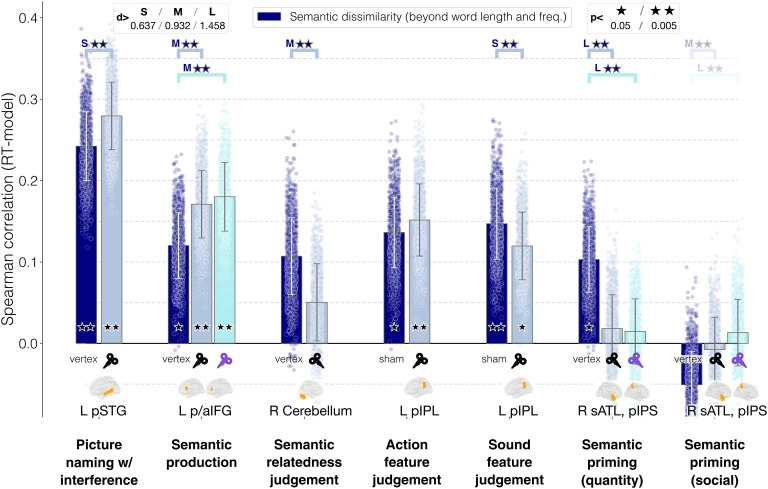
Semantic similarity results after residualization of word length and frequency. The bar heights correspond to the average Spearman correlation between Pointwise Positive Mutual Information dissimilarity and response times across all bootstrap iterations; error bars represent the standard deviation; the dots correspond to the Spearman correlation for each individual iteration.

In a final set of analyses, we tested whether semantic (dis)similarity could still explain a significant portion of variance in RTs after removing the variance explained by lower-level variables (word frequency and worth length). In this way, we probed whether the effect of TMS on semantic processing was specific to semantics and not confounded by recognition or orthographic effects (see Figure 6).

To remove the variance explainable by word frequency and length alone, we adapted a cross-validated confound removal procedure validated by Snoek et al. (2019). For each subject within every iteration of the hierarchical bootstrap subsampling procedure, we trained a linear regression model on the left-out trials, learning to predict the RTs from word length and log-transformed word frequency. Then, we predicted the RTs selected for the current subsampling iteration using the newly trained model and transformed the original RTs to the residual errors (i.e., the difference between the predicted and the true RTs; also called “residualization”; Rao et al., 2017). This procedure ensures an unbiased removal of confounding information—due to training the residualization model with a dedicated, left-out portion of the data (Snoek et al., 2019).

We report here the results only for the semantic similarity model; in the Supplementary Materials (additional Results section), it is possible to see the results also for word length and word frequency.

As expected, correlation values numerically decreased, which is likely explained by the known correlation between word length and word frequency (Piantadosi et al., 2011) as well as word frequency and semantic similarity (Burdick et al., 2018; Zhou et al., 2022). Nevertheless, all the results reported above were confirmed. For language production, semantic dissimilarity explained best RTs for word naming with interference (vertex: *ρ* = 0.241, *p* = 0.0044, *d* = 5.69, 95% CIs [0.238, 0.243]), and TMS significantly increased the way in which this type of semantic information was used, with a small effect size (*d* = −0.909, *p* = 0.0032, 95% CIs [−0.98, −0.84]). For semantic production, we replicated the original pattern of results reported above—correlations were significantly above chance in all cases (vertex: *ρ* = 0.121, *p* = 0.0085, *d* = 3.105, 95% CIs [0.119, 0.124]), and TMS again increased the correlation between semantic dissimilarity and RTs (pIFG: *d* = −1.464, *p* = 0.0032, 95% CIs [−1.55, −1.38]; aIFG: *d* = −1.425, *p* = 0.0032, 95% CIs [−1.51, −1.34]).

For semantic judgment tasks too, the semantic effect of TMS on RTs was left untouched by residualization, consistently decreasing correlation between semantic dissimilarity and RTs. After removing the variance explained by lower-level variables, correlation between semantic dissimilarity and RTs in the semantic relatedness judgment task was significantly above chance (vertex: *ρ* = 0.105, *p* = 0.05, *d* = 2.17, 95% CIs [0.102, 0.108]), as was the difference between effective stimulation of the cerebellum and vertex (*d* = 1.18, *p* = 0.0032, 95% CIs [1.1, 1.27]). For the sound and action judgment data set and the priming study, results followed the same pattern as above—TMS had a slightly detrimental effect on correlations between RTs and semantic similarities for sound feature judgments (vertex: *ρ* = 0.149, *p* = 0.0043, *d* = 3.475, 95% CIs [0.147, 0.152]) and semantic priming with quantity concept words (vertex: *ρ* = 0.099, *p* = 0.031, *d* = 2.52, 95% CIs [0.097, 0.102]), but the semantic effects remained well above significance (sound feature judgment: *d* = 0.762, *p* = 0.0032, 95% CIs [0.69, 0.83]; quantity priming, right pIPS: *d* = 2.16, *p* = 0.0032, 95% CIs [2.06, 2.28]; quantity priming, right sATL: *d* = 2.08, *p* = 0.0032, 95% CIs [1.98, 2.2]). As before residualization, semantic effects on TMS for action feature judgment did not reach significance (*d* = −0.425, *p* = 0.0032), and for social priming, semantic similarity did not show significant correlation with RTs (*ρ* = −0.051).

## DISCUSSION

In this study, we show that language models provide new insight into TMS-induced modulations of semantic cognition during word production and comprehension.

Our main finding was that semantic similarity could be used to successfully capture how RTs are modulated by NIBS in trial- and word-specific semantic processing. We found that TMS affected semantic processing at the level of individual words, as measured with the language model, across almost all areas, tasks, and data sets, with only minor exceptions (again social concept priming and action feature judgment). Overall, these findings validate our approach. Importantly, they confirm that language models, whose ability to predict brain responses to language is by now well established, can be used to capture the subtle variations in behavioral measures induced by TMS—opening up the possibility of future experiments where word-specific modulations are exploited together with NIBS.

However, importantly, two findings went against our predictions—highlighting the inherent challenges when looking at such fine-grained information using TMS. First, a significant stimulation effect was found to affect also lower-level, nonsemantic processing for at least two tasks (semantic production and quantity semantic priming). This indicates that even if the effects of TMS are intended to operate on one single, cognitive level (semantics), they can in fact spread to other levels of processing. We note that similar effects have been demonstrated at the cortical level: Despite stimulation targeting a single area, its effects can be widespread on the cortex (Beynel et al., 2020; Numssen et al., 2023; Schuler & Hartwigsen, 2025). Such converging evidence confirms the need for going beyond simple comparisons of RTs across binary conditions. What is needed is the comparison of explicit competing models, capturing trial-by-trial variation in the stimuli, whose relative ability to explain the RTs can be assessed and interpreted in the context of general theoretical models of linguistic and semantic processing (Kriegeskorte & Douglas, 2018; Lopopolo et al., 2024; Naselaris & Kay, 2015; Peelen & Downing, 2023). Second, semantic priming involving social semantic concepts could not be captured by semantic similarity; this may, however, be due to limitations in the model (see below for a discussion of this point).

Based on our results, one might ask whether the effects on semantic processing were in fact determined by lower-level variables, instead of semantic information. To test that this was not the case, we ran a post hoc analysis, removing the variance in the RTs that could be explained by word frequency and length before looking at correlations between RTs and the semantic model. The final results confirmed the significance of all the TMS effects on semantic processing that we found in the original analyses, beyond what could be explained by lower-level variables. The only difference to report is an overall numerical decrease in correlation values—which was expected since during language processing, variables at different levels tend to be correlated with each other (Burdick et al., 2018; Piantadosi et al., 2011; Zhou et al., 2022).

The second finding that went against our expectations was that effective stimulation did not always decrease the correlation between RTs and the semantic model. This finding was consistent only for language comprehension (semantic judgments) tasks. The opposite pattern (increase in correlations between the semantic model and RTs after stimulation) emerged for language production tasks, where control regions were stimulated. This implies that, during or after stimulation, the processing of a trial became more or less cognitively demanding, in proportion to the semantic similarity of its items.

While we acknowledge that it is impossible to solve this puzzle in the context of the current paper, we interpret our findings in the light of existing neural noise frameworks for TMS effects (Miniussi et al., 2013). We propose that this mechanism could be hypothesized to reflect the two sides of a possible TMS effect. TMS might either result in a sharpening of the stimulus sensitivity by adding an “optimal level of noise for task performance” (see Bergmann & Hartwigsen, 2021) and thereby increasing the correlation between RTs and a type of information. Alternatively, the TMS-induced noise may be detrimental, resulting in a desensitization to stimulus properties and a decrease in correlation. This is particularly relevant for future studies. It does not only provide a testable hypothesis regarding the functional effects of TMS on complex cognitive processes like language but also holds the promise of shedding light onto the mechanistic neural effects of the causal links behind semantic cognition.

In the following paragraphs, we discuss our findings more in depth in light of our hypotheses and previous studies, and we highlight the limitations of our approach.

### Hypothesis 1: Semantic Similarity Explains RTs in Almost All Behavioral Tasks

Our results indicate that—with the only exception of semantic priming with social concepts—semantic (dis)similarity as computed from a language model is capable of reliably capturing the cognitive effort associated with different semantic tasks, as measured in reaction times. This aligns with previous studies on semantic priming (Jones et al., 2006) and semantic relatedness judgments (Gatti et al., 2022). Nevertheless, our results move beyond theses initial findings and provide important new insight into semantic processing.

First, our results generalize to a much wider range of tasks where reaction times are used to capture semantic processing, spanning both language comprehension and production. Previous studies largely focused on comparing semantic similarity measures from language models to explicit word ratings (e.g., the model-based similarity vs. the average human similarity rating for the pair of words “concepts” and “object”; Banks et al., 2021; Connell & Ramscar, 2001; Lenci et al., 2022; Wingfield & Connell, 2022). Here, by contrast, we show that language models are able to go one step further—significantly explaining trial-by-trial RTs, a measure of implicit cognitive effort (Baayen & Milin, 2010), across a range of different tasks.

Moreover, we analyzed various data sets with an approach that strictly matched the final computation of statistics, ensuring a straightforward side-by-side interpretation of the general results. We adopted a bootstrap approach based on subsampling, providing a standardized evaluation of the amount of variance that could be explained by semantic similarity for each task. Such standardized scores are useful for planning future TMS studies on semantic processing as they offer a set of testable hypotheses with respect to the effects of TMS on semantic processing—both in terms of expected baseline values as well as directionality of the effect (e.g., increase/decrease with stimulation at a certain site).

Our results allow researchers to choose a task whose cognitive effort can be successfully captured by semantic similarity. We found that, overall, RTs in production tasks (picture–word interference, semantic production) and at least some semantic judgments (semantic relatedness, sound features) are solidly captured by a semantic similarity measure. Priming, by contrast, seemed to be slightly trickier to explain using semantic similarity; however, there may be data set–specific reasons for this pattern of results.

In particular, the only task where the correlation between language models and RTs failed to exceed the chance level was semantic priming with social concepts. Social semantic information is unique—partly because it seems to be, to some extent, segregated from general semantic knowledge (Binney & Ramsey, 2020; Olson et al., 2013) and partly because it tends to vary strongly across individuals depending on life experiences (Mazzuca et al., 2020). Consequently, it is possible that language models—whose core principle, when it comes to lexical semantics, is abstraction over many contexts of occurrence of the same word—may fail, in the absence of subject-specific information, to capture social semantic knowledge (for recent attempts at overcoming such limitations, see Bruera & Poesio, 2024; Johns, 2021).

### Hypothesis 2a: TMS Effects on Semantic Processing Are Reliably Found Across Studies, But Their Directionality Is An Open Question

We found that TMS significantly modulated the amount of variance in RTs that could be explained by semantic (dis)similarity as computed from a language model. Since language models capture a broad, generic but deep knowledge about word meaning, we interpret this as indication that TMS causally affects the way the brain makes use of semantic knowledge. Importantly, we also show that this effect went beyond what could be explained by other lower-level, nonsemantic variables.

This was true for all data sets, although not for all tasks within each data set. Namely, action feature judgments and social concept priming did not show a significant effect of stimulation on semantic processing. Regarding the latter, semantic similarity—as computed with a language model—did not seem to explain RTs at all (see previous section for a discussion). For action feature judgments, two possible explanations should be taken into account. First, the absence of a significant modulation may be explained by the way subjects “solve” the task—hence, with the way we modeled it: Semantic similarity may be a good model only for one feature (sound, where we found a significant effect), but not the other (action). Alternatively, the targeted pIPL may differentially retrieve and use sensorimotor relative to auditory information—which would reflect different processing strategies for different semantic modalities. The current analyses do not allow for a distinction between the two explanations, since we simply reanalyzed the data set and could not design ad hoc experimental manipulations to decide between the two. Nevertheless, a clear picture can be drawn at a more general level: There is an interplay between TMS effects on semantics and the individual tasks.

A more pressing open issue is the question why TMS increased the fit between language model dissimilarity and RTs for some tasks and decreased it for others. In the following, we will discuss this unexpected finding, providing post hoc explanations to motivate future studies.

A tempting explanation would be to interpret such results in light of the inhibition/excitation (also facilitation) dichotomy that is routinely used to frame TMS effects, but this has also been strongly criticized (Hartwigsen & Silvanto, 2023; Hussain & Freedberg, 2025). From this perspective, a higher correlation between RTs and semantic similarity could indicate facilitation of semantic information in the stimulated brain areas; a lower correlation would indicate, by contrast, inhibition, potentially due to an increase in noise. Our results would thus not support a consistent inhibitory effect of TMS on semantic processing across tasks and data sets—in particular, our results suggest that, in some cases (namely, comprehension tasks), stimulation led to inhibition and, in others (production tasks), to facilitation. This aligns with the ongoing debate about the unpredictable direction of TMS effects on cognitive tasks and supports the recommendation to avoid terms like “inhibitory” or “facilitatory” TMS protocol since the effect of TMS strongly depends on the specific brain state and likely interacts with a given task (see Hartwigsen & Silvanto, 2023; Hussain & Freedberg, 2025).

Such an interpretation is not warranted given our current knowledge of TMS effects on nonmotor areas (Hussain & Freedberg, 2025). Previous work has emphasized the state dependency of TMS effects, demonstrating that the same protocol can have differential effects depending on the individual brain state (e.g., task vs. rest) or level of baseline performance (see Bradley et al., 2022; Silvanto et al., 2007). Second, it is not clear whether the effect of TMS on the stimulated neurons is best described as noise injection, temporary silencing (a so-called “virtual lesion”), or something else (Ruzzoli et al., 2010). An additional issue that is relevant here is that the vast majority of studies investigating how TMS works at the level of neurons have looked at the motor cortex only. Because of the complexity of the cortex, it is not obvious that other areas will respond in the same way as the motor ones (Siebner et al., 2022). With the current analyses and without any neuroimaging data, we cannot distinguish the different possible explanations.

Nevertheless, our results show that, as expected, all semantics-related areas that were stimulated were also affected by TMS as compared to their nonstimulated state. Some of the observed differences in our results may reflect the differential role of the targeted areas in semantic processing. Stimulation of areas associated with semantic control, the left inferior frontal gyrus (IFG; Jackson, 2021) and the left pSTG (Piai et al., 2020), increased the correlation between RTs and semantic similarity, while stimulating areas not involved in semantic control but representation or integration (left pIPL, right cerebellum, right anterior temporal lobe (ATL), right pIPS) resulted in decreased correlations. A second option could be that stimulation during semantic production tasks (incidentally involving the IFG and the pSTG) has a different effect than stimulation during semantic comprehension. Differentiating between these explanations would require a controlled study that should keep all other parameters (e.g., stimulation protocol, timing, brain region) constant.

Nevertheless, our computational modeling approach complements the focus on brain areas with a discussion at the level of information processing. While such a perspective is not common in TMS studies yet (notable exceptions being reviewed in Miniussi et al., 2013), it is well established in neuroimaging and electrophysiological studies (Kriegeskorte & Bandettini, 2007; Naselaris et al., 2011). In this sense, the question is not so much whether TMS helped or impaired semantic processing. Rather, we ask whether TMS triggered, at a computational level, a process that increased (higher correlation with RTs) or decreased (lower correlation with RTs) the importance of semantic (or statistical, or orthographic) information in determining RTs.

Under such a framework, the three main observations from our analyses were as follows: First, the TMS effect on semantic information processing was statistically significant. Second, the modulatory effect interacted with individual word meaning, as captured by language models. Third, it could be isolated from other levels of linguistic processing. The increase of correlation between semantic similarity and RTs after TMS could be reformulated as follows: The easier the items were to process originally, the easier they will become after TMS, and vice versa for harder items, that become even harder as a consequence of TMS. It is evident that this is not compatible with an inhibitory/facilitatory account of TMS: This implies that TMS both helps (in the case of easier trials) and disrupts (in the case of tougher trials) processing, depending on the interaction with processing difficulty.

Another mechanistic explanation would be that TMS increased or decreased the stimulus-sensitivity of the cognitive processing at hand. In some cases (semantic production, when stimulating semantic control areas), TMS increased stimulus-sensitivity during semantic processing—sharpening the effect of semantic similarity. In other cases, by contrast, TMS made cognitive processing more generic, less affected by the semantic representations of the words. In the larger context of how exactly TMS affects information processing, this can be related to the existing literature on so-called “discrimination sensitivity” (Miniussi et al., 2013). In short, the relationship between the input sensory strength and the sensitivity in behavioral responses (e.g., in an auditory discrimination task) can be modeled by a sigmoid function, which is S-shaped: Lower strengths are compressed to 0 (i.e., not discriminated), and higher input strength is compressed to 1 (i.e., easily discriminable). Accordingly, Miniussi et al. (2013) propose that TMS shifts the sigmoid function along the *x*-axis, making it easier or tougher to discriminate at a fixed input sensory strength. We propose that in our tasks, where discrimination is not really involved, the sigmoid function may not have been shifted by TMS, but its slope may have been altered (Han & Moraga, 1995). The sigmoid function thus becomes either steeper in its central part (increasing stimulus-sensitivity, hence correlation) or, on the contrary, more horizontal (decreasing stimulus-sensitivity and correlation). Again, while it is impossible to validate whether this explanation is correct, it can motivate future studies and allow to place the effects found here in the broader context of mechanistic models of brain stimulation.

### Hypothesis 2b: TMS Effects on Semantics-Related Brain Areas Can Spread to Lower-Level Cognitive Processes

A final, important result of our analyses was unexpected. We reasoned that TMS effects should be confined to semantic processing since we included studies with semantic tasks and target areas associated with semantic processing (rather than lower-level processes). Yet, we found that, in some cases, TMS significantly affected processing of both word frequency and length.

For word frequency, this was the case only for the left IFG (see Figure 3). This is actually not surprising, since the IFG has been found to be sensitive to word frequency (e.g., Hauk et al., 2008; Sánchez et al., 2023). However, it shows that it may be premature to interpret effects on RTs as being exclusively semantic in nature. Looking deeper, however, the pattern of results could actually provide original insight with respect to the respective roles of the anterior and posterior portions of the left IFG, since they were both stimulated separately during a semantic production task.

Interestingly, the two show a clear dissociation. When looking at the cumulative effect on processing of word frequency (Figure 3), stimulation over the pIFG significantly increased correlations, while the opposite happened for the anterior part. When distinguishing between written and uttered words, the effect seems to be confined to production (see Supplementary Materials Figures C3 and C4). Consequently, we may conclude that stimulation of the left pIFG increased stimulus sensitivity for word frequency. This not only confirms its role in phonological processing but also contributes to the discussion on its functional role (cf. Fiebach et al., 2002; Klaus & Hartwigsen, 2019; Sánchez et al., 2023). Importantly, for semantics, the effect of stimulation is consistent in both portions of the IFG: On the one hand, this is in accordance with recent reviews (Jackson, 2021; Turker et al., 2023) regarding the engagement of the whole IFG in semantic processing; on the other hand, this confirms that the dissimilarity metric is sensitive to specific, selective pieces of information and allows fine-grained discrimination among different effects.

For word length, an even lower-level variable, we found that TMS unexpectedly had an effect for the left IFG, right sATL, and right pIPS. In the left IFG, the effect was consistent across but subregions, resulting in correlation decreases between RTs and word length. Again, the effect was present when either considering both the written and the uttered words’ lengths together, or the uttered word in isolation, but not when considering only the length of the written word (Supplementary Materials Figures C3 and C4). This confirms the role of the left IFG at lower linguistic levels like orthography and phonology (Indefrey, 2011; Pammer et al., 2004). Also, this effect is interestingly opposed in directionality to that for word frequency: The same stimulation can increase stimulus sensitivity for one piece of information and decrease it for another. In this sense, tracking the interactions between stimulation and computational processes can provide new insight for cognitive models of information processes during language processing (de Zubicaray, 2023).

Regarding the right sATL and the right pIPS, the effect of stimulation on processing of word length was significant only for quantity concept priming. It was found to act especially on the target word (Supplementary Materials Figures C11–C14), and it acted in different directions across the two areas (decrease in correlation between RTs and word length for the sATL, increase for the pIPS). This is relatively surprising. Both areas are usually associated with higher-cognitive functions (semantic, nonlinguistic processing for the right ATL [Lambon Ralph et al., 2017; Rice et al., 2018] and numerosity and spatial processing for the right IPS [Binkofski et al., 2016; Dehaene et al., 2005]) and only recent reports suggest they may both play a role in word processing (for the right ATL: Cope et al., 2020; for the right IPS: Santacroce et al., 2024).

Finally, the discrepancy in results across types of priming remains puzzling: One would assume that TMS should have equally affected the processing of word length, given that they were part of the same experiment. While we are not able to explain this effect, we hypothesize that this is an after-effect of our finding that semantic similarity significantly explains priming RTs only for quantity (where also word length is significantly affected by TMS) but not for social concepts: it is possible that participants used different strategies for the two, and this significantly altered the way in which information was processed across the right pIPS and the right sATL, explaining the difference in the way TMS affected them.

In short, the above discussed results suggest that lower-level and higher-level processing interact and modulatory TMS effects may not be constrained to either level, even when higher-level areas are targeted. This is not surprising: language processing in the brain is a complex mesh of interrelated processes happening concurrently, interacting with each other and where information flows in multiple directions at the same time (Balota et al., 2004; Christiansen & Chater, 2008; Hickok & Poeppel, 2007; Levelt, 1999).

In summary, our approach shows that framing the modulatory effects of TMS on semantic processing in terms of information processing—by comparing the fit between competing models and cognitive processing—can unveil linguistic processes affected by TMS at different levels. This has the potential not only to advance our understanding of the mechanistic impact of NIBS (Balota et al., 2004; Christiansen & Chater, 2008; Hickok & Poeppel, 2007; Levelt, 1999) but also to sharpen the definition of current models of language processing.

### Limitations

A first, evident limitation of our study is that, given the considerable differences between the experimental procedures of all the preexisting data sets, as well as the obvious impossibility to control experimental parameters, our partially unexpected results cannot provide a unified, coherent picture with respect to the effects of TMS at different levels of processing. Rather, we can only propose a novel methodology of analysis, built around the notion of similarity across different levels of language processing, and reveal previously unnoticed effects of brain stimulation. Therefore, while our results confirm the effectiveness of TMS as an analytic tool to study semantics in the brain and of an information-based framework that exploits language models and similarity as a powerful lens to look at the effects of TMS, it will be necessary in the future to explicitly design experiments that, by controlling the experimental parameters and employing this methodology, can provide a more comprehensive picture.

A second obvious limitation is the fact that the pattern of stimulation effects on semantics that we found, while presenting clear-cut insights (selective significant results for semantics, difference between production and comprehension and/or control/representation areas), is also at times ambiguous (conflicting effects on orthography and frequency processing, different directionalities of effects potentially due to multiple factors). We would like to point out that this could be seen as a strength, rather than a limitation of this work—because we believe that the ability to reveal inconsistencies and previously overlooked phenomena is central to advancing scientific theories and understanding. Nevertheless, we also acknowledge that our results leave multiple questions open, to be addressed systematically in future work.

Finally, a clear limitation is that, even if the concept of similarity is sufficiently high level and general to be operationalized for all three linguistic levels considered here (semantics, frequency, orthography), similarity measures differ in their actual computation across levels. Moreover, within the level of semantics, this is true also in the case of Kuhnke et al. (2020), where we used a dedicated prototype-based approach. We note that this can be related to the broader, long-standing debate on the validity of similarity as a cognitive construct (Goodman, 1972; Medin et al., 1993; Roads & Love, 2024). In this literature, it has been underlined that similarity depends on the specific context (or frame) in which it is carried out. For instance, it has been proposed that, because of the inherent qualitative differences between distinct levels of processing, it is irremediably different to compute the similarity of two words in terms of, e.g., length as opposed to their meanings. The validity of our results does not necessarily depend on specific measures of similarity or their implementation—because we simply required measures that could be good enough to capture the different levels of processing. Yet, we acknowledge that other measures—based on similarity or other constructs—could capture the different levels of linguistic processing more comprehensively. We leave this for future work that can build on the current results using similarity as the main conceptual framework.

## MATERIALS AND METHODS

### Data Sets

Included data sets were chosen based on the following five criteria: (1) TMS study with a language task, (2) including at least one TMS and one sham or other control condition, (3) a focus on lexical semantics (i.e., the meaning of individual words, not words in context), (4) reporting a significant stimulation effect on semantic processing, and (5) publicly and fully available data set according to the FAIR Open Data principles (Wilkinson et al., 2016). First, we screened the publications of a recent review (Qu et al., 2022), identifying three papers that gave full public access to the data sets (Klaus & Hartwigsen, 2019; Kuhnke et al., 2020; Piai et al., 2020). Then, we expanded our search to Google Scholar, looking for papers respecting our criteria and containing one of the following combinations: “TMS” + “semantic,” “brain stimulation” + “semantic,” “TMS” + “concepts,” and “brain stimulation” + “concepts.” We found two additional papers (Catricalà et al., 2020; Gatti et al., 2022). In the following, we provide short descriptions of the main characteristics of each data set; a comparative visual overview is also given in Figure 1 (for detailed information, please refer to the Data Sets subsection of the Supplementary Materials).

The first data set employed as a task picture naming with interference (Piai et al., 2020). TMS was applied on left mid-superior temporal gyrus to pSTG, an area reported to play an important role in semantic control, and particularly in resolving semantic interference (i.e., correctly naming a picture despite the presence of a distracting word; Jackson, 2021). Stimulation was online, consisting of burst of five pulses at 10 Hz delivered at each picture onset. TMS intensity was set to 90% of the individual motor threshold of the left primary motor hand area. The authors reported a significant facilitatory effect on RTs (i.e., lower RTs for TMS vs. vertex) for a subset of the overall trials (cases where task difficulty was higher, and both the picture and the word referred to the same concept).

The second data set was described in Klaus and Hartwigsen (2019). The experiment consisted of two production tasks—one phonological and one semantic; given our focus, we only analyzed the RTs for the semantic one. TMS was applied at two sites: pIFG and aIFG. Such regions were chosen because of their putative selective engagement for respectively phonological and semantic processing (Poldrack et al., 1999). TMS intensity was set to 90% of the motor threshold for the right-hand region of the primary motor cortex. The authors reported an inhibitory effect on semantic processing when stimulating the aIFG (i.e., higher RTs for TMS vs. vertex).

The third data set was first analyzed in Gatti et al. (2022). The task was a semantic relatedness judgment between a noun and an adjective. Online 20-Hz triple-pulse TMS was applied either at the noun or adjective onset in a counterbalanced fashion. The stimulated area was the right cerebellum, which has started to be recently associated with semantic processing (Murdoch, 2010). Results revealed that the TMS effect shifted from facilitation to inhibition in semantic processing as the similarity between nouns and adjectives increased.

The fourth data set was first presented in Kuhnke et al. (2020). There were two different but structurally identical semantic feature judgment tasks: auditory feature and action feature judgment (i.e., “Is the following concept related to sound?” and “Is the following concept related to action?”). TMS was delivered as four online 10-Hz pulses 100 ms after word onset. The brain area of interest was the pIPL, found to be consistently activated by a large number of semantic processing tasks (Jackson, 2021). The authors found a significant effect on accuracy (importantly, not on RTs) for the action feature judgment task alone.

The final data set was introduced in Catricalà et al. (2020). It tested two types of semantic priming—using primes referring to either social (i.e., “sociability”) or quantity (i.e., “immensity) concepts. There were two stimulation sites of interest: the right sATL, hypothesized to be selectively involved in social semantics, and the right pIPS, associated with numerical information and quantification. Additionally, as control site, the authors used the vertex. Double-pulse TMS at 25 Hz was delivered between prime and target words 10 ms after the blank following the prime word. Intensity was set at 100% of the motor threshold. The authors found that stimulation for the regions of interest, as opposed to vertex, reduced the priming effect for social words in both pIPS and sATL and for quantity words only in the pIPS condition.

Note that we always used the original exclusion criteria for trials and subjects reported in the individual papers. Following common procedures (Van Zandt, 2002), we transformed RTs to their logarithm before entering the analyses to ensure normality of their distributions.

### Measuring the Correlation Between Models and RTs

The goal of our study was to probe different models (semantic, frequency based, and orthography based) to find out whether the TMS effects found across five data sets and attributed originally to semantics alone, on the basis of simple contrasts of conditions (i.e., direct comparisons of RTs across stimulation conditions), were actually due to a selective semantic effect.

The intuition behind our methodology is quite straightforward: For each trial, we modeled the cognitive effort in terms of the three models (semantic similarity among the words involved, sum of word frequencies or word lengths). Then, we used Spearman correlation (*ρ*) to measure the association between trial-level RTs and each of the three models. We ran the analyses separately for each subject and then averaged the *ρ* scores across subjects, thus accounting for subject-specific differences in reaction times.

This approach is very similar to representational similarity analysis (RSA), which is widely used in cognitive neuroscience (Kriegeskorte et al., 2008). In particular, ours could be considered a special case of RSA in which, in technical terms, the reaction times are taken as the so-called “first-order similarities” for the behavioral measures. In simpler terms, we interpret reaction times during an experimental task as reflecting the similarity between two representations, capturing the result of an implicit process of comparison between two representations, happening at the cognitive level of processing (Naselaris et al., 2011). From this perspective, the final correlation values between each model and the reaction times can be interpreted as RSA correlation scores.

For each model, we then compared the correlations across effective stimulation and control conditions (sham/vertex). A significant difference among the two for a given model would indicate that the TMS effect acted on that level of cognitive processing (semantic, lexical/recognition, orthographic). As detailed above, we hypothesized that significant differences among TMS conditions should emerge only for the semantic model.

Our method brings together the advantages afforded by traditional approaches, such as univariate statistical tests (used in most of the studies from which our data sets were drawn: Catricalà et al., 2020; Klaus & Hartwigsen, 2019; Kuhnke et al., 2020; Piai et al., 2013) and linear mixed models (used in Gatti et al., 2022). First of all, as every RSA approach, it does not require fitting a model, avoiding by design the risk of overfitting (Diedrichsen & Kriegeskorte, 2017); second, hierarchical bootstrapping provides a solid way to account for both item-specific and subject-specific (also called “random”) effects (Saravanan et al., 2020); third, with the addition of a cross-validated confound removal procedure, we could obtain an unbiased measure of the specificity to semantics of the stimulation effects (in linear mixed models, this is achieved by adding additional predictors to the formula).

### Models

#### Language model semantic similarity

As a model of lexical semantics, we used so-called “language models,” also known as distributional semantics models. These are computational models that try to capture the meanings of words by abstracting them over large amounts of texts, called “corpora” (Lenci, 2018). These models can be used to answer questions that can be either theoretical—e.g., How humans acquire conceptual knowledge in the wild? (Hosseini et al., 2024; Landauer & Dumais, 1997)—or practical—e.g., Can we simulate the way humans use words in natural language for applied tasks like translation? (Bengio et al., 2003; Min et al., 2023). Although each model can differ noticeably in its implementation, they all start from a basic principle: They create vector representations for word meanings by learning from patterns of word co-occurrences in corpora. For instance, they learn that not only “wine” is similar to “glass,” because they are often found in the same sentences, but also that “wine” is similar to “beer,” because they are both frequently found in the same sentence as “glass.”

From the point of view of semantics, it has been consistently shown that such vector representations—often called “word vectors”—capture quite well the way in which humans represent the corresponding concepts (Schrimpf et al., 2021; Wingfield & Connell, 2022).

Since an extremely large number of models exists, we selected the language model that best captured behavioral responses—as measured from an independent task and data set, ensuring an unbiased evaluation.

First, we selected a number of candidate models that respected three criteria: having been previously used in the cognitive literature; differentially covering the three different types of architectures and approaches to language modeling across the years (as defined in Connell & Lynott, 2024; Lenci et al., 2022: count-based models, word embeddings, contextualized—both small and large—language models), being trained exclusively in the two languages present in our work (German and Italian). We thus restricted the set of candidates to Pointwise Positive Mutual Information (PPMI; Levy et al., 2015), fasttext (Bojanowski et al., 2017), GPT2-small (Radford et al., 2019), and Llama (Grattafiori et al., 2024).

We used the word vectors extracted from each language model to measure semantic similarity, by which we refer to the cosine similarity between two word vector semantic representations—this is the standard way of measuring semantic similarity in the field (Turney & Pantel, 2010). Conversely, when referring to “semantic dissimilarity,” we refer to 1 − semantic similarity, which we assume to proportionally reflect processing effort (the more dissimilar two representations, the more demanding the processing and the longer the reaction times). We also considered so-called “word surprisal,” a measure of conditional word probability that has been recently shown to capture to a surprising extent semantic processing (Wilcox et al., 2023). We could compute surprisal measure only for PPMI, GPT2-small, and Llama since fasttext does not allow us to do so.

Second, we singled out the language model able to best model behavioral RTs when processing semantics. We used a behavioral task, lexical decision, which seemed an ideal choice for several reasons. First, it was not used in any of the included TMS data sets, ensuring an unbiased evaluation. Second, lexical decision is commonly thought to cover multiple processing levels that are relevant here—sublexical, lexical and semantic processing (Balota et al., 2007). Finally, previous work already established that language models are able to capture the cognitive effort involved in this task (Hollis & Westbury, 2016).

We thus used publicly available reaction times data during a lexical decision task. For German, we used the data set presented in Schröter and Schroeder (2017), counting 1,152 words, and we employed only the RTs from young adults. For Italian, we used the data set published with Vergallito et al. (2020), comprising 1,121 words rated by young adults.

Finally, we computed the extent to which each model captured lexical decision times in both languages using a correlation metric. The aim was to single out the model showing the highest correlation with the lexical decision RTs for further analyses. Details of the vector extraction procedure as well as the methods and results of the evaluation are reported in the Supplementary Materials (Details and Evaluation of the Language Models section; Figures B1 and B2). These also include the full set of results for the second-best model, GPT2-small surprisal (Figures C1–C14). The model that performed better turned out to be PPMI; therefore, we used this model for all the analyses reported in the main paper.

#### Word frequency

To test whether TMS selectively affected semantic processing, we also repeated the analyses using word frequency, a variable that is not semantic in nature. By contrast, it is related to a lower-level, statistical learning effect: Words that have been encountered more often are easier to recognize (i.e., they have lower RTs in a number of tasks; see Brysbaert et al., 2017; Kuperman et al., 2024).

For word frequency, we used, as a measure of cognitive effort, the negative of the sum of the logarithm to the base 10 of the frequencies of all words involved in each trial (following Brysbaert et al., 2017). The intuition was that the less frequent the words, the bigger the cognitive effort should be; because of this inverse relation, we used the negative of the sum. For the production tasks, we summed the log-transformed frequency of the visually presented and the uttered words. For the semantic judgments tasks, we summed the log-transformed frequency of the visually presented words, which in all cases—except sound and action feature judgments—were two. For the latter case, we just took the log-transformed frequency of the word that appeared during each trial.

Summing the frequency values could lead, in principle, to missing important parts of information regarding the TMS effect—because it may have acted only on the first or the second word. To show that this was not the case and that summing the frequency values reliably captured the overall effort of each trial, we repeated the analyses using either only the first word or the second word’s log-transformed frequencies. Results are reported in the Supplementary Materials (Figures C1–C14). They confirm that, overall, the sum approach is the best frequency-based model, yielding in the vast majority of cases the highest correlation.

#### Word length

Additionally, we ran the analyses for word length, a purely orthographic variable that has consistently been shown to affect word processing (see Barton et al., 2014).

Similarly to word frequency, we assumed that the longer the words, the larger the cognitive effort should be. Again, as a measure of effort, we took the sum of all word lengths involved in each trial: for production tasks, the sum of visually presented and uttered words; for semantic judgment tasks and for sound and action feature judgments, the length of the word to be judged in each trial; for all other cases, the sum of the two visually presented words.

Likewise, we also reran the analyses using either the first word’s or the second word’s length—they are reported in the Supplementary Materials (Figures C1–C14). Again, we found that summing the lengths of all words in a trial provided the model with the best performance.

#### Hierarchical bootstrapping with subsampling

The remaining challenge was to standardize correlation scores across studies to allow easier interpretation of the results—which is not trivial, since evaluations are affected by experimental parameters like the number of subjects and trials and cannot be directly compared (Guerra et al., 2020; Hebart & Baker, 2018). To overcome this, we used hierarchical bootstrapping with subsampling, a simple but powerful nonparametric approach. This approach has been demonstrated to provide the same rate of false positives as a linear mixed-model approach, and it accounts for each of the hierarchical structure of the data set (i.e., individual subjects are resampled and evaluated separately; Saravanan et al., 2020). It relies on simulating *N* repetitions of each experiment using a fixed number of subjects and trials across all studies. In our case, *N*_iterations_ = 1,000; for each iteration, subjects and trials were randomly subsampled with fixed *N*_subjects_ = 20, *N*_trials_ = 25 from the full experiments. In practice, for each iteration in a data set—and separately for each condition and model—we sampled 20 subjects, 25 trials for each of them; computed the Spearman correlation within each subjects; and then averaged the individual scores. This allowed us to exactly match the number of subjects and trials across the five data sets (note that, as in the original papers, we only used correct trials, hence the lower number of trials available). Because of this, the resulting average correlations between each model and the RTs stem from standardized experimental conditions and thus have the same scale (see Figure 2 for a simplified visualization of the methodology used for the analyses).

#### Statistical significance testing, correction for multiple comparisons, and effect sizes

We ran two types of statistical significance tests. First, we checked whether, for each model and each TMS/control condition, the distribution of 1,000 scores obtained with hierarchical bootstrapping was significantly above or below chance (thus using a two-tailed approach). To do so, we used the procedure recommended in Saravanan et al. (2020), which is similar to the one typically used in permutation testing (Nichols & Holmes, 2002). We counted the number of iterations where the average correlation was below the value corresponding to the null hypothesis (* ρ* = 0) and divided this number by *N*_iterations_ = 1,000. This provides a one-sided *p* value, a so-called *p*_bootstrap_ (Saravanan et al., 2020)—the probability of finding a result below chance when repeating the experiment. In fact, we smoothed the *p* value by adding 1 to both sides of the division—this avoids having 0 as a resulting *p* value, as recommended in Phipson and Smyth (2010). To obtain a two-sided *p* value, we followed the implementation of the python package scipy for two-tailed permutation testing (Virtanen et al., 2020): We repeated the same operation with the inverse direction (considering average iteration values above chance), and then we took the minimum of the two and multiplied it by two, thus arriving at the *p* value. Since the *t* statistic is affected by the number of observations, which here is “artificially” set at *N*_iterations_ = 1,000, we do not report *t* values but only Cohen’s *d*, which is independent of sample size.

Second, we compared control with effective stimulation(s), separately for each data set and each model. We ran nonparametric, two-sample two-sided permutation tests. Here, we report Cohen’s *d* effect sizes for paired data.

We corrected for multiple comparisons using the false discovery rate (Benjamini & Hochberg, 1995). We used an extremely strict approach, correcting all *p* values referring to the comparisons across all TMS data sets together once (i.e., calling only one time scipy’s *false_discovery_control* function on all *p* values—including comparisons against chance and comparisons between conditions). This rigorous correction for multiple comparisons ensures keeping the risk of incurring in false positives at a minimum.

We also report 95% confidence intervals, following recent recommendations (Cumming, 2014; Hentschke & Stüttgen, 2011). As a lower bound to consider a result statistically significant, we set the threshold at *p* < 0.05; however, to avoid false positives, we complement it with thresholds for effect sizes tailored to the cognitive neuroscience literature (Szucs & Ioannidis, 2017), following recent recommendations (Correll et al., 2020; Schäfer & Schwarz, 2019). As thresholds for low, medium, and strong effects, we selected the 25th percentile (*d* = 0.637), median (*d* = 0.932), and 75th percentile (*d* = 1.458) of the effect sizes for statistically significant results reported in a recent review on effect sizes in cognitive neuroscience (Szucs & Ioannidis, 2017). These values are more stringent than generic effect size thresholds (low = 0.2, mid 0.5, high = 0.8; Calin-Jageman, 2018)—allowing to reduce the risk of false positives and highlighting which results can be confidently interpreted as reflecting an effect of TMS. To compute effect sizes and confidence intervals, we use the python package pingouin (Vallat, 2018).

## ACKNOWLEDGMENTS

We would like to thank the members of the Cognition and Plasticity lab for feedback on earlier versions of these analyses as well as the anonymous reviewers for their constructive suggestions and observations that helped us improve the paper.

## FUNDING INFORMATION

Gesa Hartwigsen, H2020 European Research Council (https://dx.doi.org/10.13039/100010663), Award ID: ERC-COG-2021-101043747. Gesa Hartwigsen, Deutsche Forschungsgemeinschaft (https://dx.doi.org/10.13039/501100001659), Award ID: HA 6314/4-2, HA 6314/10-1.

## AUTHOR CONTRIBUTIONS

**Andrea Bruera**: Conceptualization: Lead; Data curation: Lead; Formal analysis: Lead; Investigation: Lead; Methodology: Lead; Resources: Lead; Software: Lead; Validation: Lead; Visualization: Lead; Writing – original draft: Lead; Writing – review & editing: Equal. **Gesa Hartwigsen**: Conceptualization: Supporting; Funding acquisition: Lead; Project administration: Equal; Supervision: Lead; Writing – review & editing: Equal.

## DATA AND CODE AVAILABILITY STATEMENT

All the code, models, and (previously published) experimental data are available on the Open Science Framework website: https://osf.io/r3pwk.

## Supplementary Material



## TECHNICAL TERMS

Semantics: The study of the meaning of words, in two senses: the way speakers use words to refer to things and concepts, and what constitutes the meaning of a word.
TMS: An experimental technique that, by way of a magnetic field, induces a short lasting electrical current pulse into the brain, in order to stimulate superficial neurons.
Language model: A statistical approach to describe human language, that aims at capturing its statistical properties, and potentially at learning to generate language that could be taken for human-generated language.
Word frequency: The number of times a word is used in natural language, usually counted automatically from a large collection of texts (called ‘corpus’).
Orthography: The description of how, in a language, written symbols (e.g., letters) relate to the sounds that compose words, and how this can be captured by general rules.
Phonology: The description of how, in a language, sounds are used and composed in a systematic way to build larger units (e.g., words), and how this can be captured by general rules.
Prototype: In semantics, given a set of objects that would be described as belonging to the same category despite the differences among the individual exemplars (e.g., all the trees), a prototype is the individual exemplar that belongs most unequivocally to that category.
