# Supplementary material for: A Novel Approach to Map the Causal Impact of Brain Stimulation on Semantic Processing With Language Models
Source: Neurobiol Lang (Camb). 2026 May 5;7:NOL.a.244. doi: 10.1162/NOL.a.244 (PMC13171204; doi:10.1162/NOL.a.244)
Supplement: Supplementary file 1 [file nol-07-244-s001.pdf]

A novel approach to map the causal impact of brain stimulation on semantic processing with language models

Andrea Bruera[1] and Gesa Hartwigsen[1, 2]

[1]Research Group Cognition and Plasticity, Max Planck Institute for Human Cognitive and Brain Sciences, Leipzig, Germany

[2]Cognitive and Biological Psychology, Wilhelm Wundt Institute for Psychology, Leipzig University, Germany

Author Note

Andrea Bruera https://orcid.org/0000-0003-2484-2483

Gesa Hartwigsen https://orcid.org/0000-0002-8084-1330

Correspondence concerning this article should be addressed to Andrea Bruera, Max Planck Institute for Human Cognitive and Brain Sciences, Stephanstrasse 1A, 04103 Leipzig, Germany. E-mail: bruera@cbs.mpg.de

Authors report no conflict of interest.

This study was supported by the Max Planck Society and the European Research Council (ERC consolidator grant FLEXBRAIN, ERC-COG- 2021–101043747). GH was further supported by the German Research Foundation (DFG, HA 6314/4–2, HA 6314/10–1).

Appendix A

Datasets

Picture naming with interference

This dataset was originally described in Piai et al. (2020). The experiment was carried out in German. The sample size in terms of subjects was 24; each subject completed 174 trials per condition (TMS and vertex). The task was picture naming with interference. During each trial, subjects first saw a picture depicting a concrete entity, accompanied by a word that could be either matched, semantically related or unrelated to the image. Then they had to name the referent of the picture. In total, 58 pictures from 14 concrete semantic categories were used. TMS was applied on left mid-to-posterior superior temporal gyrus (pSTG), an area reported to play an important role in semantic control, and particularly in resolving semantic interference (i.e. correctly naming a picture despite the presence of a distracting word; Jackson (2021)). Stimulation was online, consisting of burst of five pulses at 10 Hz delivered at each picture onset. TMS intensity was set to 90% of the individual motor threshold of the left primary motor hand area. The authors reported a significant facilitatory effect on RTs (i.e. lower RTs for TMS vs vertex) for a subset of the overall trials (cases where task difficulty was higher, and both the picture and the word referred to the same concept).

For language models, we modelled the cognitive processing by measuring the semantic dissimilarity between the word appearing below the image and the word uttered by the subject. We assumed that these measures should index a participant's effort at carrying out the task (the larger the semantic distance between the word to be uttered and the one appearing on screen, the harder the interference, hence the task requirements).

We chose this approach because it is the most parsimonious option: first, it shares the same assumption with all other measurements of semantic similarity; second, it requires no additional assumption, because it applies to all trials, without having to specify *ad-hoc* conditions. However, when looking at the specific details of this dataset, it has two

potential shortcomings. First, this dataset contains cases where the word and the picture have the same referent - i.e. cases where the semantic dissimilarity between the two is 0. Yet, this does not imply that the expected response time is also 0. We used Spearman correlation, which transforms responses to ranks before computing the correlation. Therefore, we only assumed that cases where dissimilarity is at 0 should have a *comparatively lower rank*, in terms of response times, with respect to the other cases where dissimilarity is > 0. Also, we used scipy's implementation of Spearman correlation[1], which assigns the average rank to all tied trials (i.e., trials that have the same value: if five trials are all meant to be assigned the same, lowest rank because they contained a matching pair of word and image, then they will all be assigned a rank of 2.5). This allows to account for the number of trials whose rank is expected to be the lowest possible. Second, the original results point to a difference in directionality of the TMS effect on reaction times across related and unrelated trials: when word and picture were similar in a related trial, RTs decreased, but the opposite happened in unrelated cases, because of interference. Therefore, a similar effect might also be found in our correlation metric, despite its fundamental differences with the original paper's raw response times. To test this, we performed our analyses separately on the subset of trials that involved unrelated or related words and images, with and without the trials where the word and the image referred to the same referent. The results are reported in Figure A1. In brief, correlations decreased dramatically when the condition where the word and the image referred to the same referent was not included, and they increased slightly when considering these trials, but splitting the dataset in related and unrelated trials. Importantly, however, the directionality of the TMS effect across conditions (effective vs control stimulation) remained the same in all cases. This indicates that the best way to capture the semantic processing involved in this task is (a) considering the full set of stimuli, as it is both parsimonious and sensitive; (b) including the cases where word and image have the same

[1] https://github.com/scipy/scipy/blob/v1.16.2/scipy/stats/_mstats_basic.py

referent, that are extremely important in order to solidly capture the effect on semantic processing; (c) assuming a continuous relationship between trials in terms of semantic similarity, as opposed to hypothesizing case-specific differences. In other words, this validates our modelling approach for the data of Piai et al., 2020.

Figure A1

Validation of the chosen semantic similarity measure for the data of Piai et al., 2020, across cases. *From left to right, bars represent the correlation scores between response times and semantic dissimilarity measures (best model) for dataset-specific subsets of stimuli (unrelated and related words and images, both with and without the trials where the word and the image referred to the same object - indicated as 'same'), compared to the full set of stimuli.*

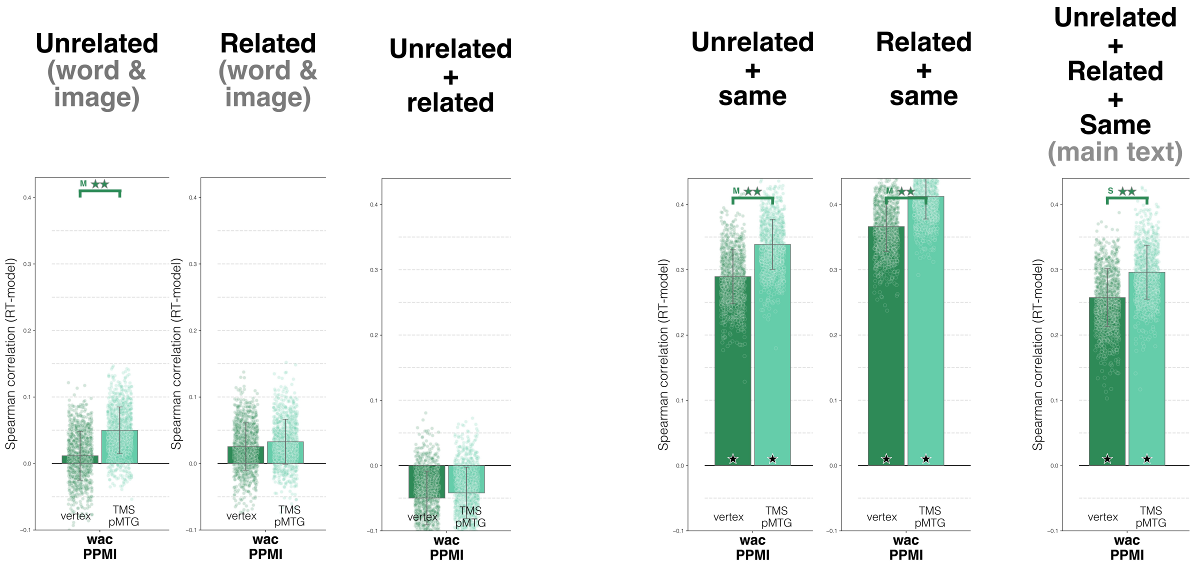

Semantic production

The second dataset was described in Klaus and Hartwigsen (2019). The experiment was carried out in German as well. The sample size of subjects was 24; each subject completed 159 trials per condition (two TMS conditions and vertex). The experiment consisted of two production tasks - one phonological and one semantic; given our focus, we only analyzed the RTs for the semantic one. Each trial consisted of a concrete word

presented on screen, and the subject's task was to produce a member of the same semantic category (e.g. input: 'cat', acceptable output: 'dog'). TMS was applied at two sites: the posterior and anterior portions of the inferior frontal gyrus (pIFG and aIFG respectively). Such regions were chosen because of their putative selective engagement for, respectively, phonological and semantic processing (Poldrack et al., 1999). TMS intensity was set to 90% of the motor threshold for the right hand region of the primary motor cortex. The authors reported an inhibitory effect on semantic processing when stimulating the aIFG (i.e. higher RTs for TMS vs vertex).

As a measure of cognitive effort for language models, we used the semantic dissimilarity between visually presented and uttered word. We assume, therefore, that, given a word as stimulus (e.g. 'cat'), it is easier to produce a member of the same category that is more similar to the stimulus (e.g. 'dog') rather than one which is less similar (e.g. 'parrot').

Semantic relatedness judgment

The third dataset was first analyzed in Gatti et al. (2022). The experiment took place in Italian. The sample size in terms of subjects was 40, with each one of them completing 70 trials per condition (TMS and vertex). During each trial participant saw two words (one noun and one adjective; e.g. 'blue', 'apricot') presented in rapid serial visual presentation, and then had to judge whether the two were semantically related. Words were semantically related in half of the trials. Online 20Hz triple-pulse TMS was applied either at the noun or adjective onset, in a counterbalanced fashion. The stimulated area was the right cerebellum, that has started to be recently associated with semantic processing (Murdoch, 2010). Results revealed that the TMS effect shifted from facilitation to inhibition in semantic processing as the similarity between nouns and adjectives increased.

Here modelling of cognitive processing was straightforward: for language models, we took the semantic dissimilarity between the adjective and the noun as the index of cognitive effort (i.e. the closer two words in meaning, the easier it is to judge them as being similar).

As with Piai et al., 2020, one might expect a different pattern of results if related and unrelated items were modelled separately (e.g. interference from lower dissimilarity in the unrelated case, and *vice-versa* for the related case). To test this, we ran the analyses separately for the unrelated and related sets of stimuli. Results are reported in Figure A2. In brief, using the whole set of stimuli again improved results (i.e. correlation scores increased), and the directionality of the effect (decrease in correlation after effective stimulation) remained the same, even when looking at specific subsets of the stimuli. This provides an empirical validation of our modelling approach to the dataset of Gatti et al., 2022.

Figure A2

Validation of the chosen semantic similarity measure for the data of Gatti et al., 2022, across cases. *From left to right, bars represent the correlation scores between response times and semantic dissimilarity measures (best model) for dataset-specific subsets of stimuli (unrelated and related adjectives and nouns), compared to the full set of stimuli.*

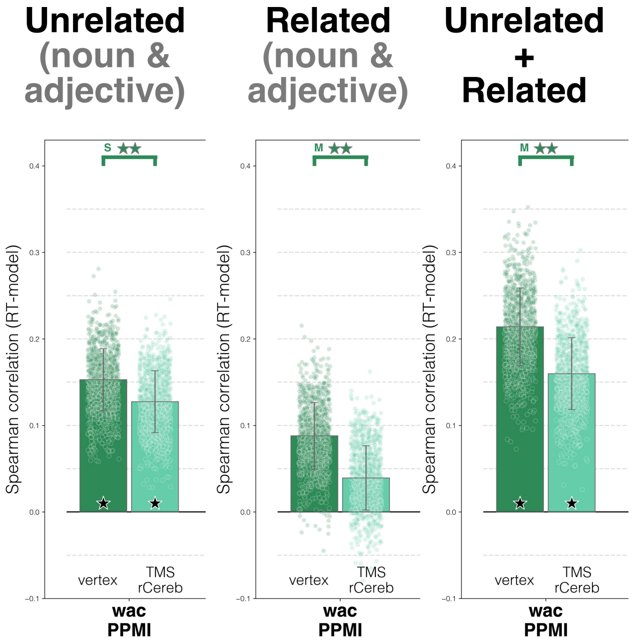

Sound and action feature judgment

This dataset was first presented in Kuhnke et al., 2020. The language of the experiment was once again German. The number of subjects was 26, that completed 52 trials per task and condition (sound and action feature judgment; TMS and sham). As anticipated, there were two different, but structurally identical tasks: auditory feature and action feature judgment (i.e. 'is the following concept related to sound?', 'is the following concept related to action?'). The experiment was divided in blocks, whose task was previously declared (i.e. sound or action). In each trial, a word referring to a concrete object was presented and participants had to indicate whether it was related to the block's feature. TMS was delivered as four online 10Hz pulses 100ms after word onset. The brain area of interest was the posterior inferior parietal lobe (pIPL), found to be consistently activated by a large number of semantic processing tasks (Jackson, 2021). The authors found a significant effect on accuracy (importantly, not on RTs) for the action feature judgment task alone.

Here, since each trial involved only one word (i.e. the word to be judged as either sound-related or action-related), we used a different approach, inspired by the concept of semantic prototype (E. H. Rosch, 1973). We started from the original intuition of E. Rosch and Mervis (1975): the difficulty of categorizing a concept, as measured in reaction times, depends on the similarity between the concept and the prototype for the category to be judged. For instance, for the category 'furniture', 'chair' is easier to judge as belonging to 'furniture' than 'radio'. This is because the underlying process would correspond to trying to match the concept and the prototype, and the closer the match -i.e. the similarity- between the two, the easier it is to provide the answer. We extend by analogy this reasoning to our case. We assume that the effort (i.e. response times) when judging whether a concept was sound- or action- related can be modelled in terms of semantic similarity to a sound- and action- prototype (i.e. the centroid, or average representation, of the concepts referring to something that makes sound and can be interacted with the

hands).

We created such prototype by averaging the word vectors for the words in the set of stimuli that were both sound- and action- related. This has been shown to be the best way to produce a prototype with language models (Westera et al., 2021). Then, for each trial, we modelled the underlying cognitive processing by measuring the semantic dissimilarity between the trial's word and the sound-and-action prototype (something similar, although without the *ad-hoc* creation of a prototype vector, had been done in Connell and Ramscar (2001)).

We would like to underline that, compared to all other datasets, here we took a special approach – involving the creation of a prototype vector to obtain a measure of semantic processing effort. However, when linking semantic dissimilarity and response times, the exact same assumption as for all other datasets was made: lower semantic dissimilarity should be associated with lower response times.

Semantic priming

The final dataset was introduced in Catricalà et al. (2020). It was run in Italian, testing two types of semantic priming - using primes referring to either social (i.e. 'sociability') or quantity (i.e. 'immensity') concepts. Each trial involved a judgment task, whose response time was measured and analyzed. Subjects were asked whether the target word was either social or quantity-related; successful semantic priming should lead to reduced reaction times when the target word was preceded by its category as a prime. Following the original paper, we ran the analyses on the subset of subjects for which the original results were reported - i.e. 22 subjects for social and 20 for quantity concept priming. There were two stimulation sites of interest, the right superior anterior temporal lobe (sATL) - hypothesized to be selectively involved in social semantics - and the right intraparietal sulcus (pIPS) - associated with numerical information and quantification. Additionally, as control site the authors used the vertex. For each condition, there were 56 trials for each priming type (social and quantity), involving 28 congruent (e.g. social prime,

social target) and 28 incongruent (e.g. social prime, quantity target) trials. Double-pulse TMS at 25Hz was delivered between prime and target words, 10ms after the blank following the prime word. Intensity was set at 100% of the motor threshold. The authors found that stimulation for the regions of interest, as opposed to vertex, reduced the priming effect for social words in both pIPS and sATL, and for quantity words only in the pIPS condition.

To model the cognitive processing carried out by participants, we used the well-established approach introduced in Jones et al. (2006) - consisting of measuring the semantic dissimilarity between prime and target words. The assumption was that a lower dissimilarity (higher similarity) between prime and target should make the task easier, hence reducing reaction times.

Appendix B

Details and evaluation of the language models

We report here the fundamental details of the procedure used to create and extract vectors and surprisal scores from the PPMI language model, since it is a model created from scratch. For the pretrained models (fasttext, GPT2-small, Llama) we only report the vector extraction and surprisal computation procedures; for training details see the original papers.

PPMI model creation. We follow standard practice in the field (Kiela & Clark, 2014; Lenci et al., 2022; Levy et al., 2015). We use the Wacky wide web corpora (Baroni et al., 2009), that are available in both Italian and German and are the most commonly used datasets in the cognitive literature (Gatti et al., 2020; Lenci et al., 2022; Mandera et al., 2017). For German, we used sDeWac, a cleaned version of the original DeWac corpus, counting around 900M tokens (Faaß & Eckart, 2013). For Italian, we used the basic ItWac corpus, counting around 1.5B tokens.

We compute co-occurrences among content words (nouns, verbs, adjectives, adverbs) in each of the corpora within a symmetrical window of 10 words around each word. Then, we transform the counts according to the PPMI procedure (Levy et al., 2015). This provides us with a square matrix, where each line corresponds to the PPMI-reweighted co-occurrences for word x with all other words - this constitutes also, in practical terms, the PPMI word vector. Since the dimensionality of the word vectors plays a key role in the amount of semantic information contained in them (Kiela & Clark, 2014), we sort the column words by their absolute frequency, and extract word vectors in increasingly bigger dimensionality, from 100 dimensions to 500000, following the recommendations Kiela and Clark (2014) (i.e. the initial 100 dimensions correspond to the 100 most common words in the corpus, and the final 500000 dimensions correspond to the 500000 most common words).

We follow different strategies to compute **word similarity** depending on the family

(static vs contextualized):

- **Static LMs (fasttext and PPMI)**: we extract the previously created word vectors for each word; then compute cosine similarities among each pair of words x and y appearing in a trial. For PPMI we repeat this procedure multiple times, each time increasing the dimensionality (i.e. the number of words included in the columns of the PPMI-transformed co-occurence matrix) up until a limit of 500000 words, sorted them by absolute frequency in the corpus, as recommended in Kiela and Clark (2014);
- **Contextualized LMs (GPT2-small, Llama)**: we experiment with two methodologies typically used in the literature - extracting the LM's activations for the word in isolation, and so-called representation pooling (i.e. encoding 10 sentences where a word is mentioned, then averaging the activations at each layer for the subword tokens of the relevant word - Bommasani et al. (2020) and Vulić et al. (2020)). We found that the first option provided better results, so we extracted the hidden activations across all layers using the words in isolation. Then, separately for each layer, we computed the cosine similarities among each pair of words x and y appearing in a trial.

Similarly for **word surprisal** (note that fasttext does not allow to compute word surprisal):

- **Static LM (PPMI)**: we use, as is standard in the field, a n-gram measure of association between words - i.e. the negative logarithm of the probability of finding word y in linear order after word x in the Wac corpus, considering at most $n = 5$ words after x - technically, we thus measure 5-gram word surprisal (de Varda et al., 2024; Smith & Levy, 2013).
- **Contextualized LM (GPT2-small, Llama)**: First, we encode with the LM word x followed by word y. Then, we use the most common implementation of word

surprisal in the field (Nair & Resnik, 2023), corresponding to the negative logarithm of the sum of the probabilities for each subword token composing word y.

We report here the results for the evaluation of the language models, that we carried out on datasets involving an independent behavioural task involving semantics (lexical decision), so as to find out in an unbiased way the best model to capture how cognitive effort is reflected in RTs during semantic processing. We ran the analyses both in Italian and German; in both cases, the best performing model is PPMI, both in Italian and German - that reaches a *plateau* in performance at a dimnesionality of around 100000.

For German, we used the dataset presented in Schröter and Schroeder (2017), counting 1152 words, and we employed only the response times from young adults. For Italian, we used the dataset published with (Vergallito et al., 2020), comprising 1121 words rated by young adults.

Figure B1

Model evaluation with lexical decision RTs in Italian. The x axis corresponds to vector dimensionality for PPMI, and to layer $* 10000$ *for contextualized language models (e.g. for* $layer = 12$*,* $x = 120000$*).*

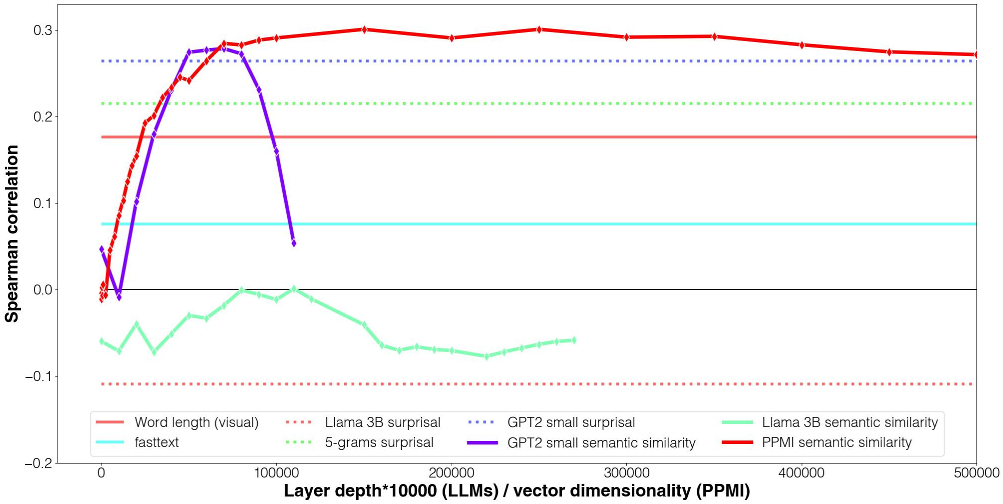

Figure B2

Model evaluation with lexical decision RTs in German. The x axis corresponds to vector dimensionality for PPMI, and to layer $*$ 10000 *for contextualized language models (e.g. for layer* $= 12$, $x = 120000$*).*

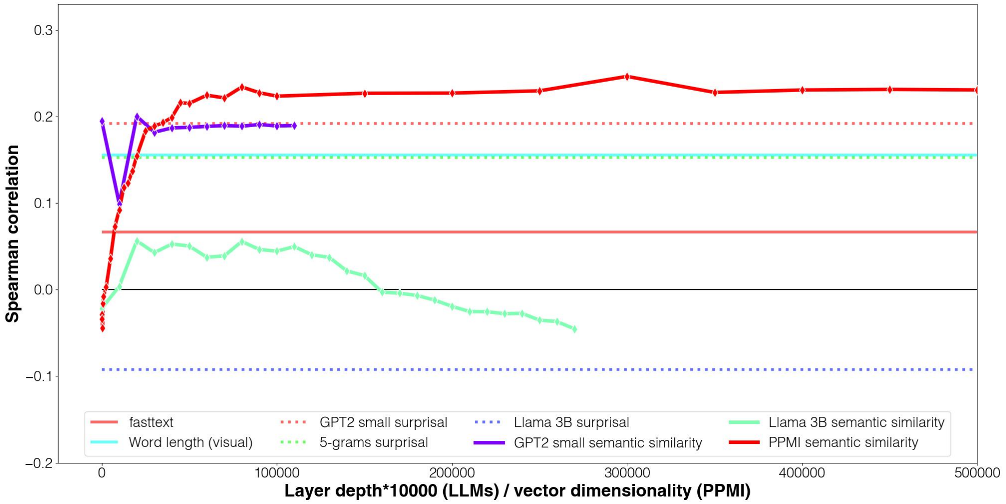

Appendix C

Additional results

In the following we complement the results reported in the main text by reporting additional results for:

- the second-best model, i.e. **word surprisal values extracted from GPT2-small**;
- **word length, separately for the first and the second word** appearing in each trial (with the exception of semantic feature judgment, where there was only one word appearing);
- **word frequency, separately for the first and the second word** appearing in each trial (with the exception of semantic feature judgment, where there was only one word appearing);

Additionally, we report the full set of results **before and after removing the variance explainable by word length and word frequency** with residualization.

First we report the plots; then the table containing the full set of statistics, both when running statistical tests against the null hypothesis and when comparing across conditions.

Figure C1

Additional results for ***picture naming with interference, no residualization***

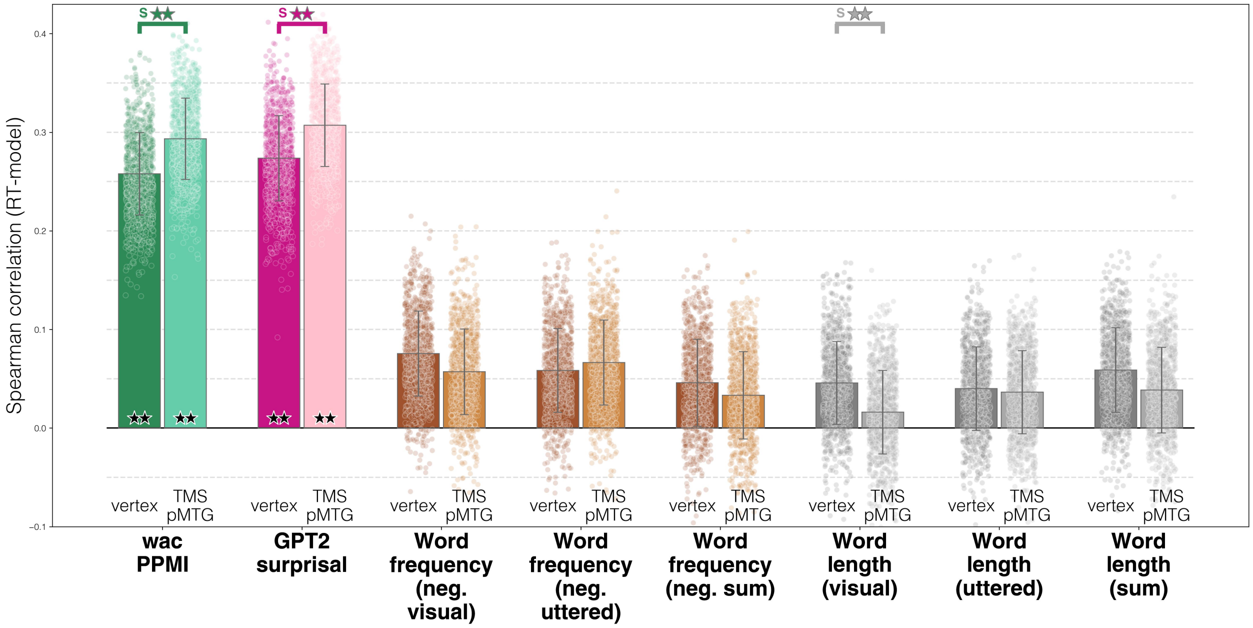

Figure C2

Additional results for ***picture naming with interference, after residualization*** *of word length and frequency*

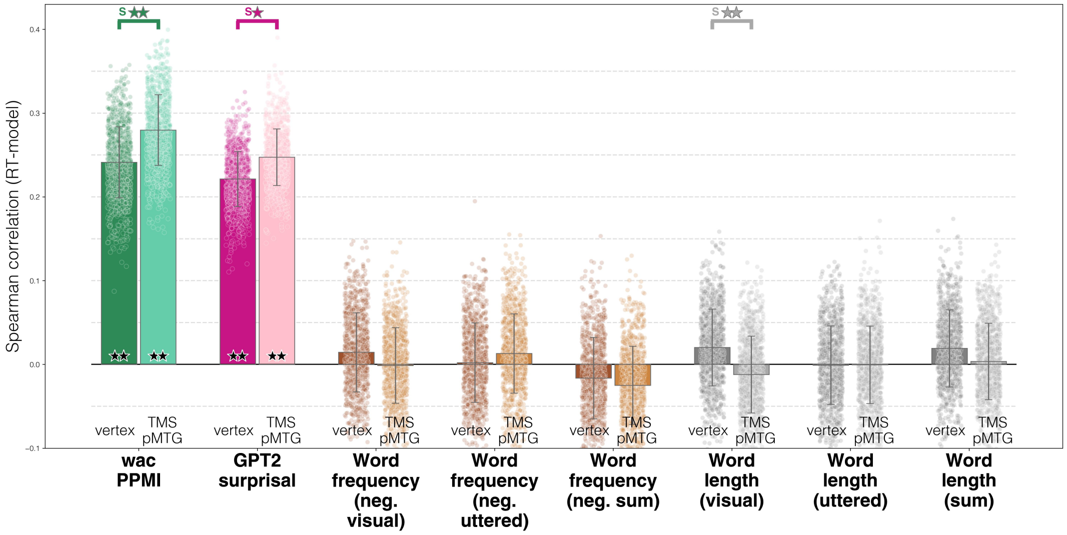

Figure C3

Additional results for ***semantic production, no residualization***

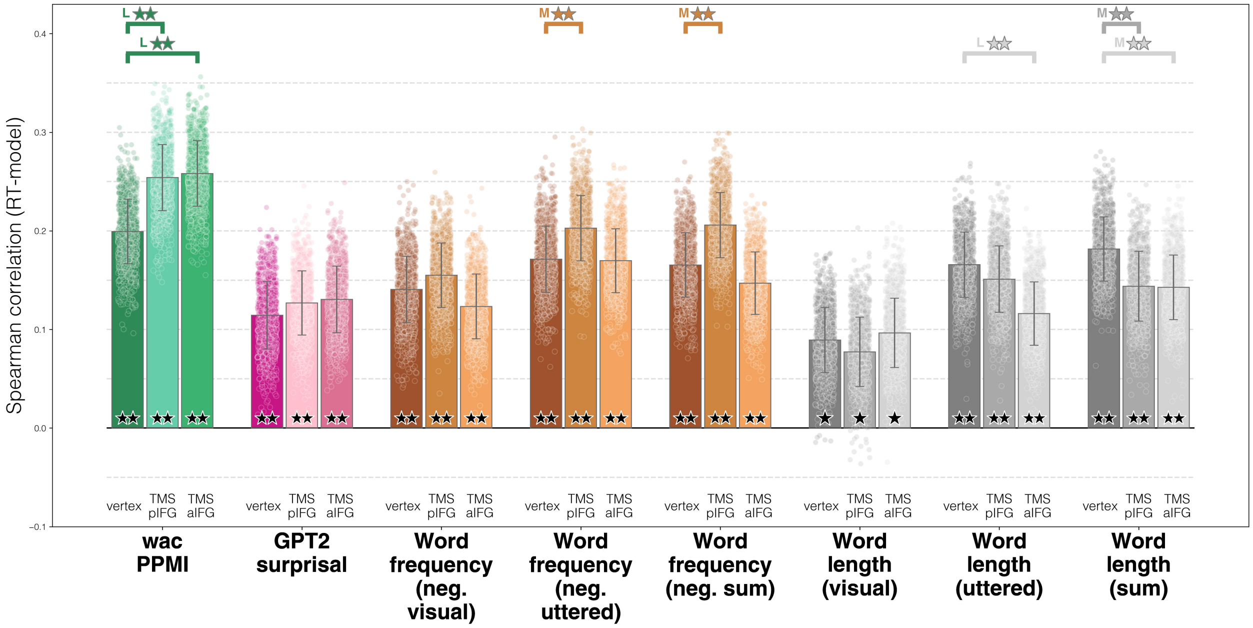

Figure C4

Additional results for ***semantic production, after residualization*** *of word length and frequency*

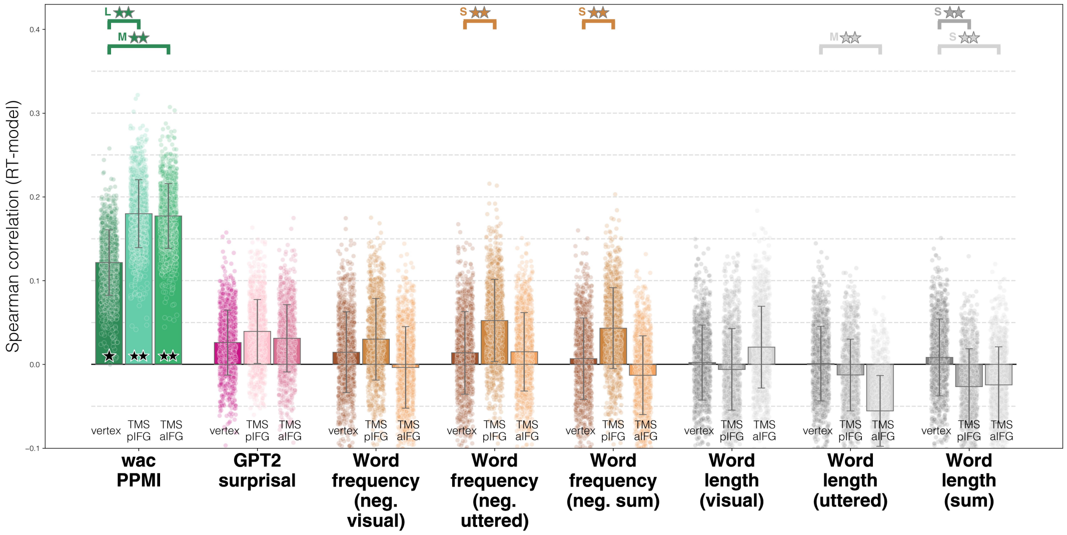

Figure C5

Additional results for ***semantic relatedness judgment, no residualization***

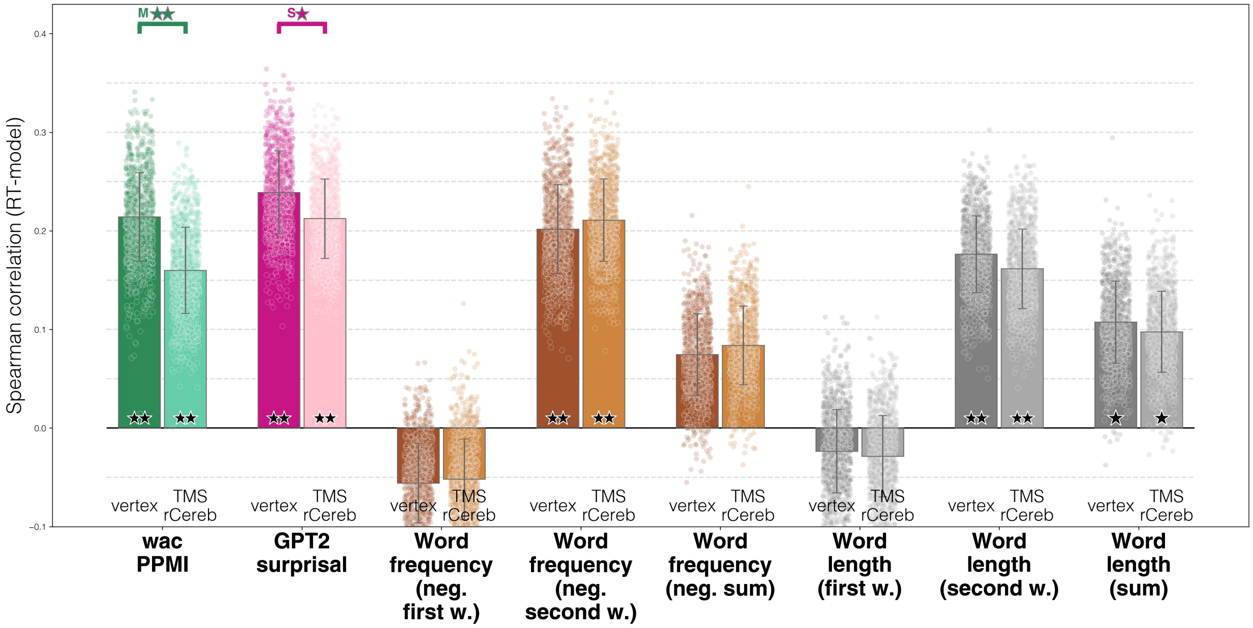

Figure C6

Additional results for ***semantic relatedness judgment, after residualization*** *of word length and frequency*

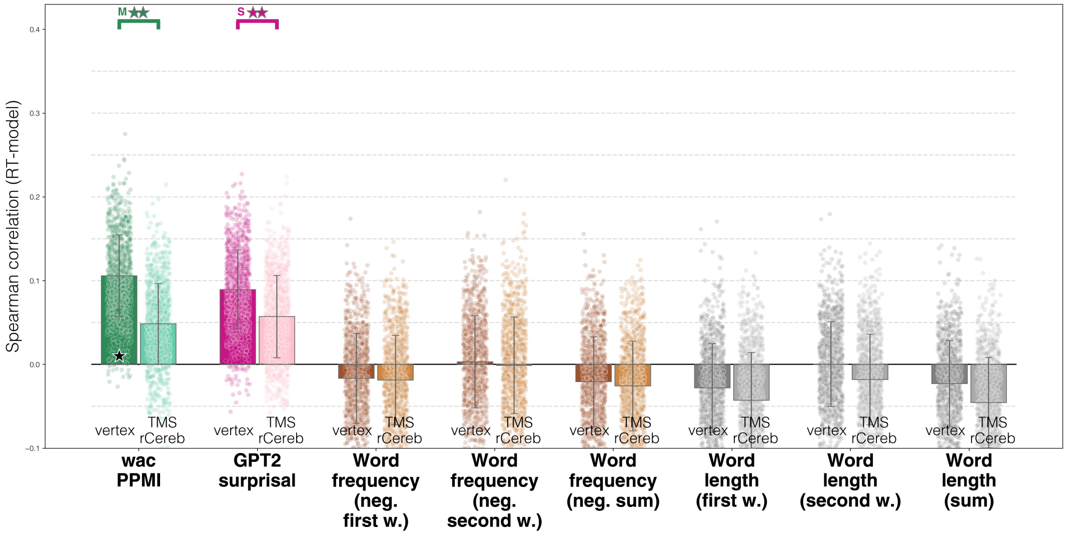

Figure C7

Additional results for ***action feature judgment, no residualization***

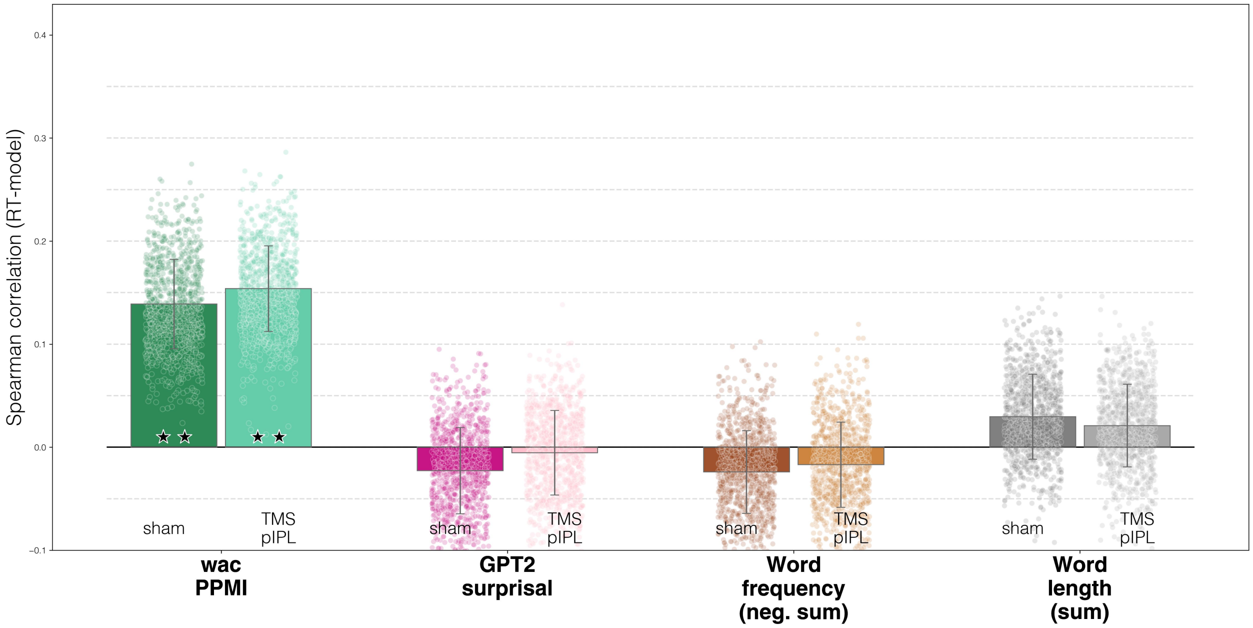

Figure C8

Additional results for action feature judgment, after residualization of word length and frequency

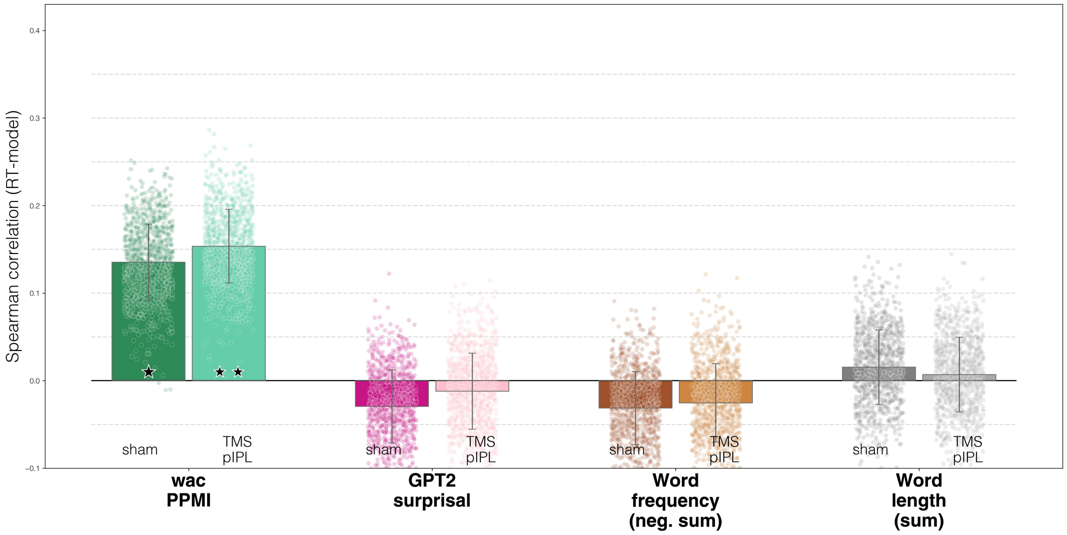

Figure C9

Additional results for ***sound feature judgment, no residualization***

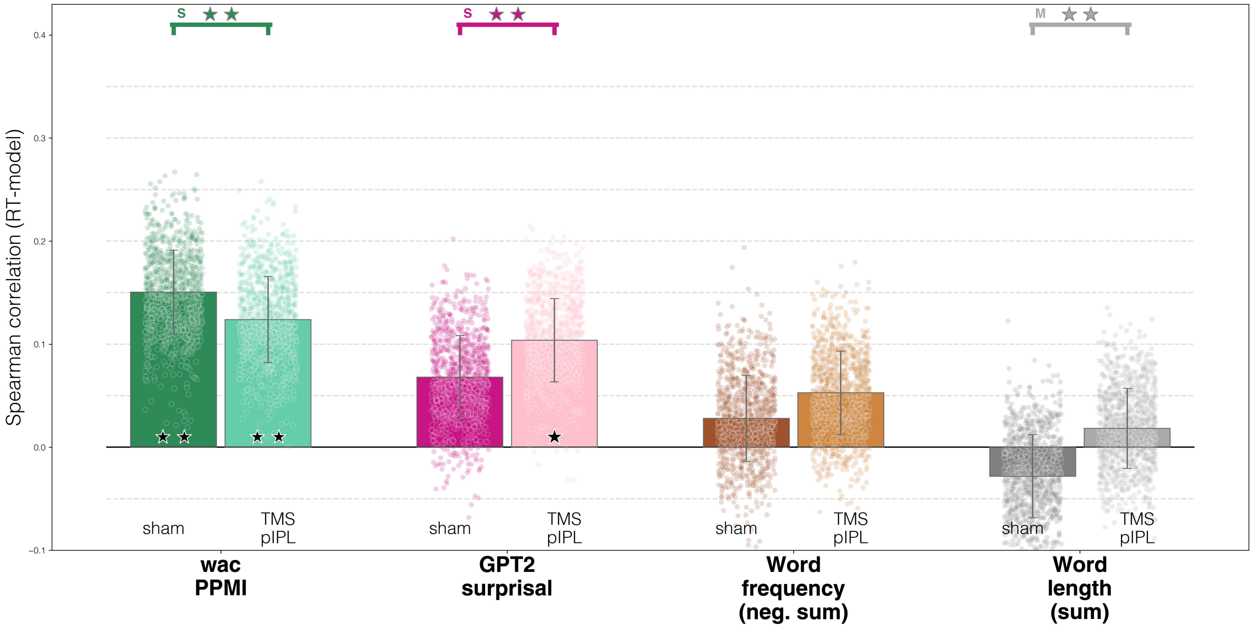

Figure C10

Additional results for ***sound feature judgment, after residualization*** *of word length and frequency*

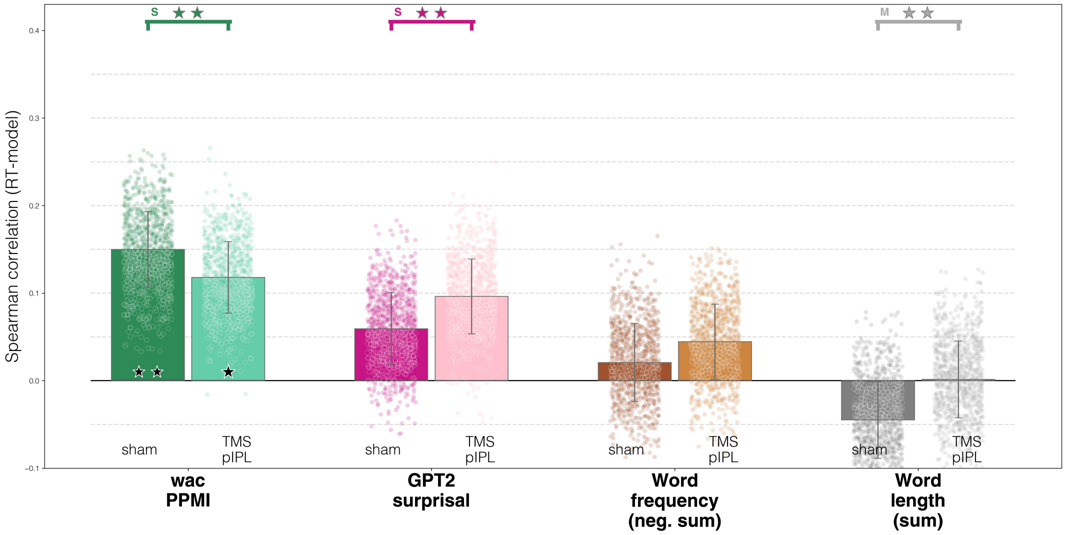

Figure C11

Additional results for ***quantity semantic priming, no residualization***

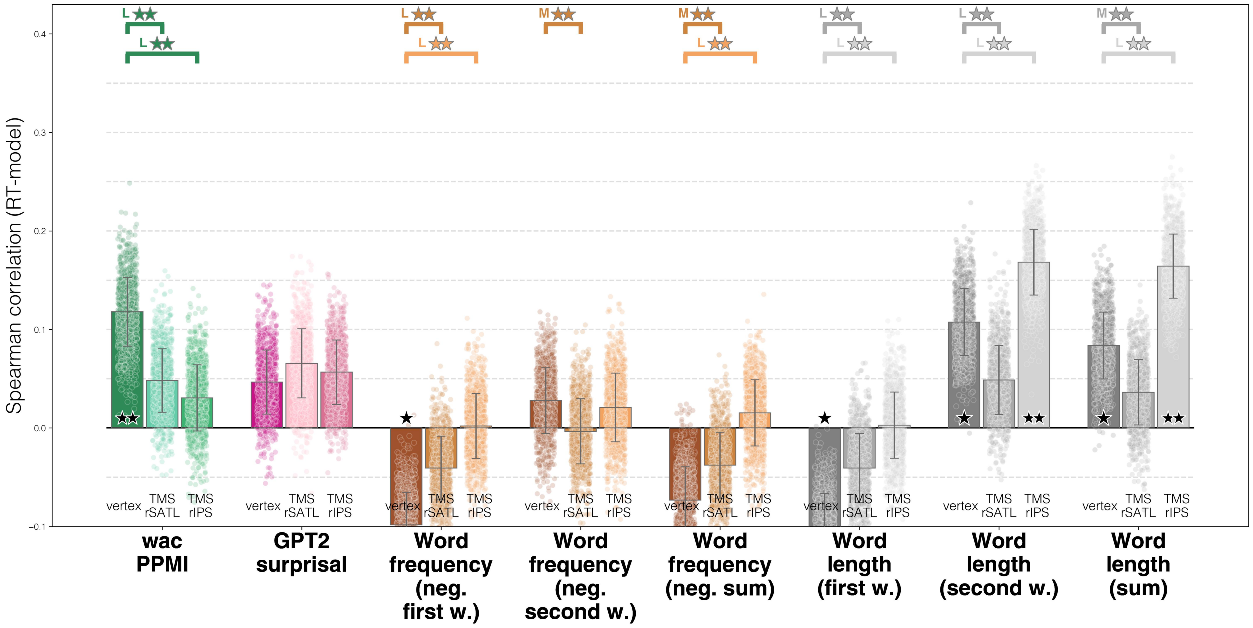

Figure C12

Additional results for ***quantity semantic priming, after residualization*** *of word length and frequency*

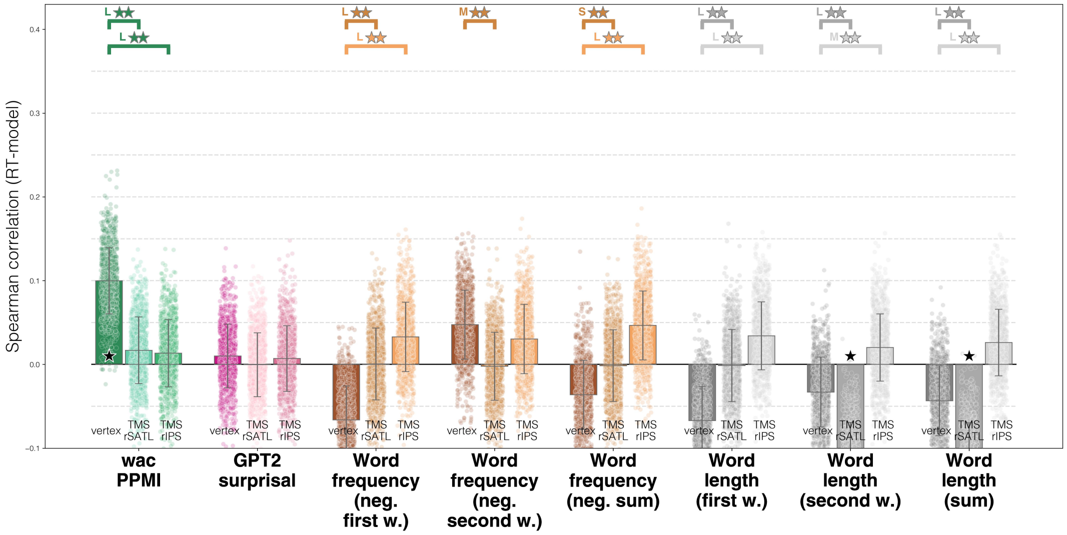

Figure C13

Additional results for ***social semantic priming, no residualization***

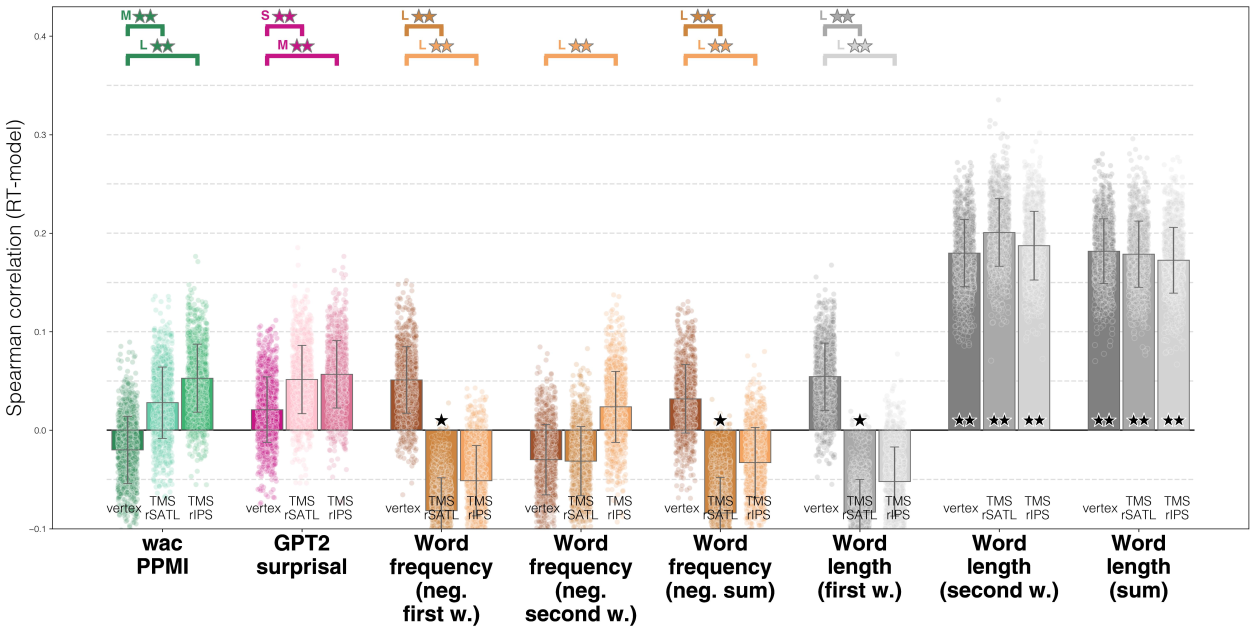

Figure C14

Additional results for ***social semantic priming, after residualization*** *of word length and frequency*

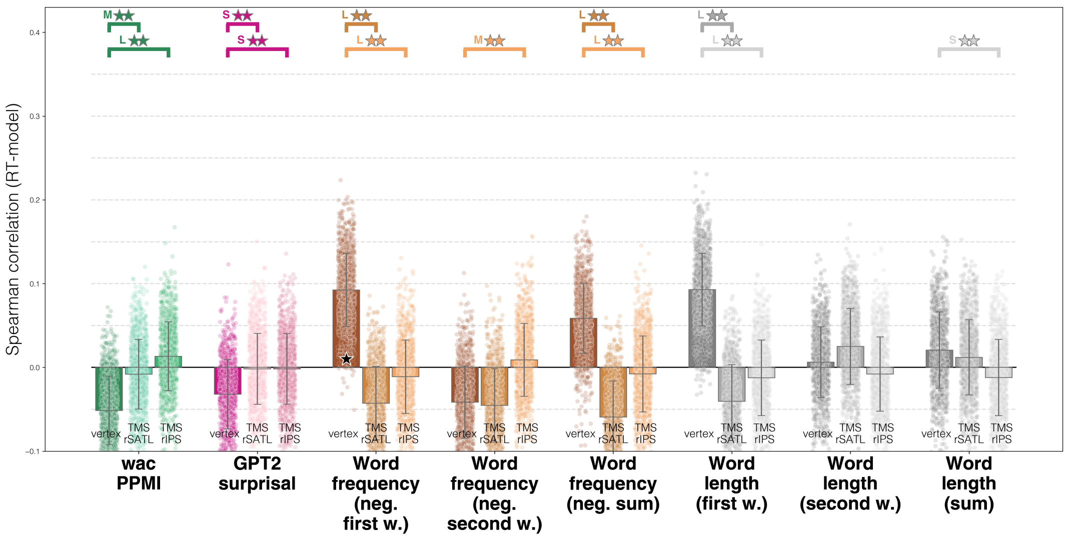

Figure C15

Full stats against null hypothesis #1

wac_corrected_baseline_p-vals

approach	dataset	condition	model	ci_min	ci_max	perms_avg	t_value	raw_p	fdr_corrected_p
**residualize**	action feature judgment	sham	wac$PPMI	0.13273035005277492	0.13810149630542504	0.13541592317909998	3.1252792	0.007992007992007992	0.01658881658881659
**residualize**	action feature judgment	TMS$pIPL	wac$PPMI	0.1509876363664787	0.15620928676952126	0.15359846156799997	3.646407	0.001998001998001998	0.004434871486894609
**residualize**	action feature judgment	sham	GPT2$surprisal	-0.03197886104601806	-0.026809831353341935	-0.02939434619968	-0.7049216	0.4895104895104895 3	0.635040635040635
**residualize**	action feature judgment	TMS$pIPL	GPT2$surprisal	-0.014826750295980295	-0.009441018812075704	-0.012133884554028	-0.2792809	0.7812187812187812	0.8797302404340527
**residualize**	action feature judgment	sham	Word$frequency$(neg. sum)	-0.03382661740840826	-0.028671344492551737	-0.03124898095048	-0.7513984	0.45554445554445555	0.6032036928588652
**residualize**	action feature judgment	TMS$pIPL	Word$frequency$(neg. sum)	-0.02811532092368997	-0.022572096280310025	-0.025343708601999998	-0.5667529	0.5794205794205795	0.7200566423867395
**residualize**	action feature judgment	sham	Word$length$(sum)	0.01272262706054926	0.01798966235871874	0.015356144709634	0.36141154	0.7272727272727273	0.8361458900381057
**residualize**	action feature judgment	TMS$pIPL	Word$length$(sum)	0.004325394698081103	0.009617005630650897	0.006971200164366	0.16330734	0.8771228771228772	0.9382038574239131
**residualize**	sound feature judgment	sham	wac$PPMI	0.1472392683415169	0.1525868087224831	0.149913038532	3.475133	0.001998001998001998	0.004434871486894609
**residualize**	sound feature judgment	TMS$pIPL	wac$PPMI	0.11531709208800323	0.12038129273999679	0.117849192414	2.8847077	0.005994005994005994	0.012577586348078151
**residualize**	sound feature judgment	sham	GPT2$surprisal	0.05685610062960739	0.061949899363288624	0.059402999996448005	1.4456152	0.15984015984015984	0.26118562288775055
**residualize**	sound feature judgment	TMS$pIPL	GPT2$surprisal	0.093618348575507	0.09891634377929301	0.0962673461774	2.2524414	0.03196803196803197	0.060175118998648414
**residualize**	sound feature judgment	sham	Word$frequency$(neg. sum)	0.017996978983021786	0.023523695494218212	0.02076033723862	0.46564326	0.6713286713286714	0.7932006455083379
**residualize**	sound feature judgment	TMS$pIPL	Word$frequency$(neg. sum)	0.0419279448459499	0.04723615442189008	0.04458204963391999	1.0411134	0.3116883116883117	0.45682561713096065
**residualize**	sound feature judgment	sham	Word$length$(sum)	-0.0474856123218358	-0.04204492715554419	-0.044765269738689994	-1.0199379	0.2997002997002997	0.4460655623446321
**residualize**	sound feature judgment	TMS$pIPL	Word$length$(sum)	-0.0013294755257670435	0.004098274853055042	0.0013843996636439995	0.03161751	0.961038961038961	0.9928894634776987
**residualize**	semantic production	vertex	wac$PPMI	0.23867014488767593	0.24392662732032402	0.24129838610399998	5.690432	0.001998001998001998	0.004434871486894609
**residualize**	semantic production	TMS$pMTG	wac$PPMI	0.27714958980993226	0.2823709095700678	0.27976024969	6.6418915	0.001998001998001998	0.004434871486894609
**residualize**	semantic production	vertex	GPT2$surprisal	0.2192624519882775	0.22333151538972248	0.221296983689	6.741664	0.001998001998001998	0.004434871486894609
**residualize**	semantic production	TMS$pMTG	GPT2$surprisal	0.24519890940392397	0.249393639276076	0.24729627433999998	7.3080187	0.001998001998001998	0.004434871486894609
**residualize**	semantic production	vertex	Word$frequency$(neg.$visual)	0.011436118250680966	0.01729718585689904	0.014366652053790003	0.30385396	0.7592407592407593	0.8625693832794424
**residualize**	semantic production	TMS$pMTG	Word$frequency$(neg.$visual)	-0.004073407553298247	0.001508356384014446	-0.0012825255846419003	-0.028482664	0.997002997002997	0.999000999000999
**residualize**	semantic production	vertex	Word$frequency$(neg.$uttered)	-0.0008915719090691189	0.004994741391407119	0.002051584741169	0.043204814	0.999000999000999	0.999000999000999
**residualize**	semantic production	TMS$pMTG	Word$frequency$(neg.$uttered)	0.010044247406808003	0.015906947446931996	0.01297559742687	0.2743568	0.7872127872127872	0.8813111087163565
**residualize**	semantic production	vertex	Word$frequency$(neg. sum)	-0.019348039555024257	-0.013328129433779739	-0.016338084494401998	-0.33643195	0.7132867132867133	0.8271368271368271
**residualize**	semantic production	TMS$pMTG	Word$frequency$(neg. sum)	-0.027831845827637952	-0.022056173217266044	-0.024944009522451998	-0.5353647	0.5754245754245755	0.7197493060685244
**residualize**	semantic production	vertex	Word$length$(visual)	0.017328107745561563	0.023017240116498434	0.020172673931029998	0.43954515	0.6413586413586414	0.7744708122066614
**residualize**	semantic production	TMS$pMTG	Word$length$(visual)	-0.014964218441954962	-0.009283221456625036	-0.012123719949289999	-0.26454368	0.7992007992007992	0.889191733122369
**residualize**	semantic production	vertex	Word$length$(uttered)	-0.003915274594184674	0.0019015427392846745	-0.0010068659274499998	-0.021457165	0.967032967032967	0.9928894634776987
**residualize**	semantic production	TMS$pMTG	Word$length$(uttered)	-0.0034460588055185816	0.002288989059288581	-0.0005785348731150001	-0.012504853	0.999000999000999	0.999000999000999
**residualize**	semantic production	vertex	Word$length$(sum)	0.016320592377508415	0.02203599284968158	0.019178292613594997	0.4159579	0.6813186813186813	0.802534888424459
**residualize**	semantic production	TMS$pMTG	Word$length$(sum)	0.0005727890597155994	0.006233046053084401	0.0034029175564000004	0.07452489	0.9590409590409591	0.9928894634776987
**residualize**	semantic production	vertex	wac$PPMI	0.11916446935949149	0.12401763117272852	0.12159105026611	3.1057243	0.003996003996003996	0.008524808524808525
**residualize**	semantic production	TMS$pIFG	wac$PPMI	0.17744067887496587	0.18246170522903413	0.179951192052	4.4427137	0.001998001998001998	0.004434871486894609
**residualize**	semantic production	TMS$aIFG	wac$PPMI	0.17471296155728303	0.17950762451271698	0.177110293035	4.579012	0.001998001998001998	0.004434871486894609
**residualize**	semantic production	vertex	GPT2$surprisal	0.02364578712804944	0.02844877757446656	0.026047282351258	0.6722593	0.4775224775224775	0.6284500482452701
**residualize**	semantic production	TMS$pIFG	GPT2$surprisal	0.036960763721558314	0.04168738997197768	0.039324076846767995	1.0313199	0.31368631368631367	0.4580058724545416
**residualize**	semantic production	TMS$aIFG	GPT2$surprisal	0.028653212906043748	0.03362325744873626	0.031138235177390002	0.77664006	0.4115884115884116	0.56245533825605
**residualize**	semantic production	vertex	Word$frequency$(neg.$visual)	0.011580702452916062	0.017565297510363932	0.014572999981639998	0.30185634	0.7512487512487512	0.8585700014271442
**residualize**	semantic production	TMS$pIFG	Word$frequency$(neg.$visual)	0.02699660777339641	0.033028776618959595	0.030012692196178	0.61676186	0.5714285714285714	0.7170868347338936
**residualize**	picture naming w/ interf	TMS$aIFG	Word$frequency$(neg.$visual)	-0.006670940764647392	-0.0006305977429686069	-0.0036507692538079992	-0.07492191	0.9630369630369631	0.9928894634776987
**residualize**	picture naming w/ interf	vertex	Word$frequency$(neg.$uttered)	0.010758929497687742	0.016875091339284255	0.013817010418485999	0.2800407	0.7772227772227772	0.8778045483927838
**residualize**	picture naming w/ interf	TMS$pIFG	Word$frequency$(neg.$uttered)	0.0493105962767819	0.055407993671608105	0.052359294974195	1.0644747	0.30569430569430567	0.4514869745638976
**residualize**	picture naming w/ interf	TMS$aIFG	Word$frequency$(neg.$uttered)	0.01214727942510075	0.017947868010407254	0.015047573717754002	0.32157367	0.7692307692307693	0.8713410483321988
**residualize**	picture naming w/ interf	vertex	Word$frequency$(neg. sum)	0.00384178150359154	0.00985523392872846	0.00684850771616	0.14117512	0.8811188811188811	0.9398601398601399
**residualize**	picture naming w/ interf	TMS$pIFG	Word$frequency$(neg. sum)	0.04028361456872661	0.04627169292149339	0.04327765374511	0.8959057	0.3436563436563437	0.49610539836103745
**residualize**	picture naming w/ interf	TMS$aIFG	Word$frequency$(neg. sum)	-0.015815705051216777	-0.009983728479323224	-0.01289971676527	-0.2741893	0.8091908091908092	0.8951048951048951
**residualize**	picture naming w/ interf	vertex	Word$length$(visual)	-0.0005054527004422792	0.005042607364558279	0.002268577332058	0.05068722	0.977022977022977	0.9971990873847638
**residualize**	picture naming w/ interf	TMS$pIFG	Word$length$(visual)	-0.008980651595468223	-0.0029404161712717784	-0.00596053388337	-0.122325614	0.8891108891108891	0.9431452525375177
**residualize**	picture naming w/ interf	TMS$aIFG	Word$length$(visual)	0.01766255230190745	0.023711842077332554	0.02068719718962	0.4239194	0.6713286713286714	0.7932006455083379
**residualize**	picture naming w/ interf	vertex	Word$length$(uttered)	-0.0018225136312936853	0.0037086887251536853	0.00094308754693	0.02113579	0.983016983016983	0.9980099056616207
**residualize**	picture naming w/ interf	TMS$pIFG	Word$length$(uttered)	-0.015332912442824625	-0.010019063465792774	-0.0126759879543087	-0.29570502	0.7172827172827173	0.8271368271368271
**residualize**	picture naming w/ interf	TMS$aIFG	Word$length$(uttered)	-0.05810153134810279	-0.05286834076873521	-0.055484936058419	-1.3143003	0.17182817182817184	0.2772353696723445
**residualize**	picture naming w/ interf	vertex	Word$length$(sum)	0.005485512408303747	0.011172503912596252	0.00832900816045	0.1815502	0.8691308691308691	0.9374894768153195
**residualize**	picture naming w/ interf	TMS$pIFG	Word$length$(sum)	-0.029281450321697156	-0.023689109967482844	-0.02648528014459	-0.5870797	0.5774225774225774	0.7199034731502264
**residualize**	picture naming w/ interf	TMS$aIFG	Word$length$(sum)	-0.02730895082574263	-0.021676174598797376	-0.024492562712270002	-0.5390112	0.6053946053946054	0.7427205382476949
**residualize**	semantic relatedness judg.	vertex	wac$PPMI	0.09737563774312052	0.10227828516239948	0.09982696145276	2.5240808	0.015984015984015984	0.031638464628155345
**residualize**	semantic relatedness judg.	TMS$rSATL	wac$PPMI	0.014400392862609222	0.01933445320523078	0.01686742303392	0.42377016	0.6593406593406593	0.7862944508907243
**residualize**	semantic relatedness judg.	TMS$rIPS	wac$PPMI	0.010897132950253135	0.015871482315822867	0.013384307633038	0.3335383	0.7152847152847153	0.8271368271368271
**residualize**	semantic relatedness judg.	vertex	GPT2$surprisal	0.007676171916353 7115	0.01238744359043029	0.010031807753392	0.2639533	0.8011988011988012	0.889191733122369
**residualize**	semantic relatedness judg.	TMS$rSATL	GPT2$surprisal	-0.0027069789605097894	0.0020183635087897894	-0.00034430772586	-0.009032329	0.985014985014985	0.9980099056616207
**residualize**	semantic relatedness judg.	TMS$rIPS	GPT2$surprisal	0.004583020778536543	0.009433748487355455	0.007008384632945999	0.17910063	0.8771228771228772	0.9382038574239131
**residualize**	semantic relatedness judg.	vertex	Word$frequency$(neg.$first w.)	-0.06873087309522996	-0.06370768047597	-0.06621927678559998	-1.6341453	0.1038961038961039	0.18052535699594524
**residualize**	semantic relatedness judg.	TMS$rSATL	Word$frequency$(neg.$first w.)	-0.0020415911329527145	0.0032769870535927148	0.0006176979603200001	0.014396821	0.9790209790209791	0.9971990873847638
**residualize**	semantic relatedness judg.	TMS$rIPS	Word$frequency$(neg.$first w.)	0.03027162105222912	0.035416841223050874	0.03284423113764	0.79130006	0.43156843156843155	0.5814816762185183
**residualize**	semantic relatedness judg.	vertex	Word$frequency$(neg.$second v	0.044857933534515795	0.0499670138814402	0.047412473707978	1.1503658	0.25374625374625376	0.3897542457542458
**residualize**	semantic relatedness judg.	TMS$rSATL	Word$frequency$(neg.$second v	-0.0047728510580919295	0.0002573039956799278	-0.002257773531206001	-0.055639736	0.951048951048951	0.9928894634776987
**residualize**	semantic relatedness judg.	TMS$rIPS	Word$frequency$(neg.$second v	0.027775293169256567	0.032900675227443436	0.03033798419835	0.73374736	0.47952047952047955	0.6284500482452701
**residualize**	semantic relatedness judg.	vertex	Word$frequency$(neg. sum)	-0.0388932219774186	-0.03377785479734139	-0.03633553838738	-0.8805232	0.3516483516483517	0.5019812900853793
**residualize**	semantic relatedness judg.	TMS$rSATL	Word$frequency$(neg. sum)	-0.0041436231559999395	0.0011637768989679388	-0.0014899231285160006	-0.034799106	0.993006993006993	0.999000999000999
**residualize**	semantic relatedness judg.	TMS$rIPS	Word$frequency$(neg. sum)	0.04400295302741514	0.04909796996778486	0.0465504614976	1.1325684	0.25374625374625376	0.3897542457542458
**residualize**	semantic relatedness judg.	vertex	Word$length$(first w.)	-0.0695981500507601	-0.0645523989035499	-0.067075274477155	-1.6478691	0.0999000999000999	0.1751672984549697
**residualize**	quantity priming	TMS$rSATL	Word$length$(first w.)	-0.004045863213305059	0.0012902861500777585	-0.00137778853161365	-0.03200669	0.993006993006993	0.999000999000999
**residualize**	quantity priming	TMS$rIPS	Word$length$(first w.)	0.03166836696353712	0.03669373369844888	0.034181050330993	0.84314793	0.4095904095904096	0.56245533825605
**residualize**	quantity priming	vertex	Word$length$(second w.)	-0.035568396223889964	-0.03040044897112203	-0.032984422597505995	-0.7911828	0.44955044955044954	0.6014891032312635
**residualize**	quantity priming	TMS$rSATL	Word$length$(second w.)	-0.11381632711703205	-0.10861082603396793	-0.11121357657549999	-2.6483865	0.00999000999000999	0.02062453675356901
**residualize**	quantity priming	TMS$rIPS	Word$length$(second w.)	0.017676552635144276	0.022661053107395725	0.02016880287127	0.501585	0.6173826173826173	0.7526188097616668
**residualize**	quantity priming	vertex	Word$length$(sum)	-0.04590751501987191	-0.040736166670720074	-0.04332184084529599	-1.0384585	0.3096903096903097	0.45563631770528323
**residualize**	quantity priming	TMS$rSATL	Word$length$(sum)	-0.11241354504449201	-0.10742886124090799	-0.1099212031427	-2.7335682	0.005994005994005994	0.012577586348078151
**residualize**	quantity priming	TMS$rIPS	Word$length$(sum)	0.023668688321565247	0.028598383346587757	0.0261335358340765	0.65714955	0.5074925074925075	0.6517629527662973
**residualize**	quantity priming	vertex	wac$PPMI	-0.05392054309795559	-0.048855302952688404	-0.051387923025321997	-1.2576133	0.2177822177822178	0.33995273019663264
**residualize**	quantity priming	TMS$rSATL	wac$PPMI	-0.01075317475123462	-0.005595055812701381	-0.008174115281968	-0.1964425	0.8571428571428571	0.9297820823244551

Figure C16

Full stats against null hypothesis #2

**residualize**	quantity priming	TMS$rIPS	wac$PPMI	0.010735788363755591	0.01582828853898041	0.013282038451368	0.32331043	0.7572427572427572	0.8625693832794424
**residualize**	quantity priming	vertex	GPT2$surprisal	-0.03459309716719036	-0.029450056653521628	-0.032021576910355995	-0.7718072	0.4515484515484515	0.602064602064602
**residualize**	quantity priming	TMS$rSATL	GPT2$surprisal	-0.004360061788224574	0.0008922155685685755	-0.0017339231098279996	-0.040923078	0.961038961038961	0.9928894634776987
**residualize**	quantity priming	TMS$rIPS	GPT2$surprisal	-0.004453116986822619	0.0007661939791146195	-0.0018434615038539998	-0.043783147	0.967032967032967	0.9928894634776987
**residualize**	quantity priming	vertex	Word$frequency$(neg.$first w.)	0.08976844546507684	0.09517497189416316	0.09247170867962	2.120199	0.025974025974025976	0.04937636620804938
**residualize**	quantity priming	TMS$rSATL	Word$frequency$(neg.$first w.)	-0.04541254160069664	-0.03997224480128356	-0.04269239320099	-0.9727785	0.35564435564435565	0.5058053058053058
**residualize**	quantity priming	TMS$rIPS	Word$frequency$(neg.$first w.)	-0.013896473592703424	-0.008455554297984575	-0.011176013945344	-0.2546248	0.8151848151848152	0.8969368740142379
**residualize**	quantity priming	vertex	Word$frequency$(neg.$second v	-0.04428675632599907	-0.03894128523395293	-0.041614020779976	-0.9650277	0.3336663336663337	0.48533284896921264
**residualize**	quantity priming	TMS$rSATL	Word$frequency$(neg.$second v	-0.048014712672837634	-0.04258715717014236	-0.045300934921489996	-1.0346392	0.3016983016983017	0.447305590162733
**residualize**	quantity priming	TMS$rIPS	Word$frequency$(neg.$second v	0.006257972262176051	0.011656434421587949	0.008957203341882	0.20567827	0.8471528471528471	0.9241667423485606
**residualize**	quantity priming	vertex	Word$frequency$(neg. sum)	0.056043131963376534	0.06121732956774747	0.058630230765562	1.4046388	0.16183816183816183	0.2633298904485345
**residualize**	quantity priming	TMS$rSATL	Word$frequency$(neg. sum)	-0.062034361174638425	-0.056683946601697566	-0.059359153888167995	-1.3752649	0.1918081918081918	0.30561969151180773
**residualize**	quantity priming	TMS$rIPS	Word$frequency$(neg. sum)	-0.010568781516001113	-0.004946602948378887	-0.00775769223219	-0.1710464	0.8891108891108891	0.9431452525375177
**residualize**	quantity priming	vertex	Word$length$(first w.)	0.09017197883279522	0.0955495910938048	0.09286078496330001	2.1405675	0.03796203796203796	0.0700837623914547
**residualize**	social priming	TMS$rSATL	Word$length$(first w.)	-0.04319361334143902	-0.03777079582230096	-0.04048220458186999	-0.9253909	0.3696303696303696	0.5180221238615399
**residualize**	social priming	TMS$rIPS	Word$length$(first w.)	-0.015205148430231876	-0.009617150304028127	-0.012411149367130002	-0.2753226	0.7912087912087912	0.8832098134423715
**residualize**	social priming	vertex	Word$length$(second w.)	0.00362653651972273	0.00886891721709927	0.006247726868411	0.14773369	0.8671328671328671	0.9374894768153195
**residualize**	social priming	TMS$rSATL	Word$length$(second w.)	0.02220741338106162	0.02783495288933838	0.0250211831352	0.551157	0.5914085914085914	0.7325835454867713
**residualize**	social priming	TMS$rIPS	Word$length$(second w.)	-0.010679999479707027	-0.005166970014772974	-0.00792348474724	-0.17816073	0.8431568431568431	0.9224279993510762
**residualize**	social priming	vertex	Word$length$(sum)	0.017882027883683115	0.023521781322476884	0.02070190460308	0.45502603	0.6513486513486514	0.779183433389041
**residualize**	social priming	TMS$rSATL	Word$length$(sum)	0.009242823489074712	0.014818820570767291	0.012030822029921001	0.26746002	0.8211788211788211	0.9009504780933352
**residualize**	social priming	TMS$rIPS	Word$length$(sum)	-0.014917739523594238	-0.009268069568333776	-0.012092904545966	-0.26533446	0.8111888111888111	0.8951048951048951
**residualize**	social priming	vertex	wac$PPMI	0.10282989340095816	0.10886579906552182	0.10584784623323999	2.1738305	0.029970029970029972	0.056692076396509895
**residualize**	social priming	TMS$rCereb	wac$PPMI	0.045590302823483725	0.05153662042293228	0.048563461623208	1.0123894	0.2957042957042957	0.4418305429978581
**residualize**	social priming	vertex	GPT2$surprisal	0.0864007198157467	0.0922494340303333	0.08932507692304	1.893211	0.07192807192807193	0.12846688195525405
**residualize**	social priming	TMS$rCereb	GPT2$surprisal	0.054013835220523373	0.060110472506096635	0.057062153863310004	1.1602294	0.26573426573426573	0.4033278973990437
**residualize**	social priming	vertex	Word$frequency$(neg.$first w.)	-0.02000060958391925	-0.013363741964010752	-0.016682175773965	-0.31158432	0.7872127872127872	0.8813111087163565
**residualize**	social priming	TMS$rCereb	Word$frequency$(neg.$first w.)	-0.02204485560365208	-0.01541962975425792	-0.018732242678955	-0.35048962	0.6953046953046953	0.8140152530396433
**residualize**	social priming	vertex	Word$frequency$(neg.$second v	-0.0003419037186107093	0.006456576705150708	0.0030573364932699994	0.05574648	0.955044955044955	0.9928894634776987
**residualize**	social priming	TMS$rCereb	Word$frequency$(neg.$second v	-0.004656304271186223	0.002511736561078223	-0.001072283855054	-0.018543664	0.971028971028971	0.9943336663336663
**residualize**	social priming	vertex	Word$frequency$(neg. sum)	-0.023988342602234335	-0.01734296508359366	-0.020665653842913998	-0.38549215	0.7072927072927073	0.825356461720098
**residualize**	social priming	TMS$rCereb	Word$frequency$(neg. sum)	-0.02907396646149088	-0.022438264404009124	-0.025756115432750002	-0.48114896	0.6193806193806194	0.7526650564625249
**residualize**	social priming	vertex	Word$length$(first w.)	-0.03135834084930286	-0.024805142509397134	-0.02808174167935	-0.53119844	0.5934065934065934	0.7326949577753435
**residualize**	social priming	TMS$rCereb	Word$length$(first w.)	-0.04643848054598549	-0.039353903034594524	-0.04289619179029001	-0.75056934	0.45554445554445555	0.6032036928588652
**residualize**	social priming	vertex	Word$length$(second w.)	-0.0029467557448207226	0.0033339304977367226	0.00019358737645800006	0.0038208135	0.955044955044955	0.9928894634776987
**residualize**	social priming	TMS$rCereb	Word$length$(second w.)	-0.021431047930577846	-0.014712926201622158	-0.0180719870661	-0.33346027	0.7312687312687313	0.8382304262901278
**residualize**	social priming	vertex	Word$length$(sum)	-0.02611899407549437	-0.019741682523043234	-0.022930338299268802	-0.44571668	0.6413586413586414	0.7744708122066614
**residualize**	social priming	TMS$rCereb	Word$length$(sum)	-0.04897628056527877	-0.04229328641266123	-0.04563478348897	-0.84646887	0.3916083916083916	0.5428795031683119
**bootstrap**	action feature judgment	sham	wac$PPMI	0.13621006057939017	0.1415787173126098	0.13889438894599998	3.2070453	0.001998001998001998	0.004434871486894609
**bootstrap**	action feature judgment	TMS$pIPL	wac$PPMI	0.15132528498928258	0.15647269266771743	0.1538989888285	3.7062376	0.001998001998001998	0.004434871486894609
**bootstrap**	action feature judgment	sham	GPT2$surprisal	-0.02534921291238661	-0.02015612248313339	-0.02275266769776	-0.54311585	0.6013986013986014	0.7401828940290478
**bootstrap**	action feature judgment	TMS$pIPL	GPT2$surprisal	-0.0079983357321367	-0.0029108538480733	-0.005454594790105001	-0.13290633	0.945054945054945	0.9928894634776987
**bootstrap**	action feature judgment	sham	Word$frequency$(neg. sum)	-0.026564843616154396	-0.021589842662397607	-0.024077343139276	-0.5999312	0.5274725274725275	0.6729217626227594
**bootstrap**	action feature judgment	TMS$pIPL	Word$frequency$(neg. sum)	-0.019556821215321585	-0.01442363533801842	-0.016990228276670002	-0.41029698	0.6693306693306693	0.7932006455083379
**bootstrap**	action feature judgment	sham	Word$length$(sum)	0.02710856372533762	0.03222253213814439	0.029665547931741004	0.7190853	0.4835164835164835	0.6293909480350158
**bootstrap**	action feature judgment	TMS$pIPL	Word$length$(sum)	0.01844271669757884	0.023419054076717162	0.020930885387148	0.52139133	0.6073926073926074	0.7427986026712141
**bootstrap**	sound feature judgment	sham	wac$PPMI	0.14791605745089853	0.15295766817310152	0.15043686281200003	3.6988866	0.001998001998001998	0.004434871486894609
**bootstrap**	sound feature judgment	TMS$pIPL	wac$PPMI	0.12125233207669366	0.12643299107150635	0.1238426615741	2.9632707	0.001998001998001998	0.004434871486894609
**bootstrap**	sound feature judgment	sham	GPT2$surprisal	0.06548176012186796	0.070493707187952	0.06798773365490998	1.6815515	0.10589410589410589	0.18316818316818315
**bootstrap**	sound feature judgment	TMS$pIPL	GPT2$surprisal	0.10133042466556437	0.10634825264983565	0.10383933865770001	2.565265	0.015984015984015984	0.031638464628155345
**bootstrap**	sound feature judgment	sham	Word$frequency$(neg. sum)	0.025446357939132972	0.03062670938062703	0.02803653365988	0.67088974	0.48151848151848153	0.6289220983098535
**bootstrap**	sound feature judgment	TMS$pIPL	Word$frequency$(neg. sum)	0.050323081418289096	0.055329827323670915	0.052826454370980006	1.3079224	0.21578421578421578	0.3382087300454647
**bootstrap**	sound feature judgment	sham	Word$length$(sum)	-0.030704284848596705	-0.025702551231083295	-0.02820341803984	-0.698984	0.4915084915084915	0.6354857263948173
**bootstrap**	sound feature judgment	TMS$pIPL	Word$length$(sum)	0.015846102766849463	0.020660731751238535	0.018253417259044	0.46996707	0.6453546453546454	0.7768532408030842
**bootstrap**	semantic production	vertex	wac$PPMI	0.1974002213814982	0.20146394554650182	0.19943208346400002	6.083547	0.001998001998001998	0.004434871486894609
**bootstrap**	semantic production	TMS$pIFG	wac$PPMI	0.2519363674814221	0.2560825630385779	0.25400946526	7.594272	0.001998001998001998	0.004434871486894609
**bootstrap**	semantic production	TMS$aIFG	wac$PPMI	0.2561078151681851	0.2602299920118148	0.25816890358999994	7.763603	0.001998001998001998	0.004434871486894609
**bootstrap**	semantic production	vertex	GPT2$surprisal	0.11226177595320365	0.11651412727879633	0.11438795161599999	3.3345497	0.001998001998001998	0.004434871486894609
**bootstrap**	semantic production	TMS$pIFG	GPT2$surprisal	0.12485166672157993	0.12889486424242003	0.12687326548199998	3.8898351	0.001998001998001998	0.004434871486894609
**bootstrap**	semantic production	TMS$aIFG	GPT2$surprisal	0.12844066711784885	0.13263438982215114	0.13053752847	3.858529	0.001998001998001998	0.004434871486894609
**bootstrap**	semantic production	vertex	Word$frequency$(neg.$visual)	0.13856070569384743	0.14272699104015257	0.140643848367	4.184637	0.001998001998001998	0.004434871486894609
**bootstrap**	semantic production	TMS$pIFG	Word$frequency$(neg.$visual)	0.1530134601336137	0.15707334819438631	0.155043404164	4.733968	0.001998001998001998	0.004434871486894609
**bootstrap**	semantic production	TMS$aIFG	Word$frequency$(neg.$visual)	0.12132379870508363	0.12539086531891638	0.123357332012	3.7598433	0.001998001998001998	0.004434871486894609
**bootstrap**	semantic production	vertex	Word$frequency$(neg.$uttered)	0.16908104243171063	0.1732603184422894	0.171170680437	5.0770845	0.001998001998001998	0.004434871486894609
**bootstrap**	semantic production	TMS$pIFG	Word$frequency$(neg.$uttered)	0.20065342357122612	0.20476944426877386	0.20271143392	6.1050158	0.001998001998001998	0.004434871486894609
**bootstrap**	semantic production	TMS$aIFG	Word$frequency$(neg.$uttered)	0.16769819773023967	0.17171494104776028	0.16970656938899997	5.2373385	0.001998001998001998	0.004434871486894609
**bootstrap**	semantic production	vertex	Word$frequency$(neg. sum)	0.1633285740074006	0.1673759932285994	0.165352283618	5.064285	0.001998001998001998	0.004434871486894609
**bootstrap**	semantic production	TMS$pIFG	Word$frequency$(neg. sum)	0.20377116855551203	0.207880892260488	0.20582603040800002	6.208315	0.001998001998001998	0.004434871486894609
**bootstrap**	semantic production	TMS$aIFG	Word$frequency$(neg. sum)	0.1448986267982632	0.14882739416573676	0.14686301048199998	4.6338525	0.001998001998001998	0.004434871486894609
**bootstrap**	semantic production	vertex	Word$length$(visual)	0.08723162706770204	0.09132765910849797	0.0892796430881	2.7019367	0.011988011988011988	0.024228403175771594
**bootstrap**	semantic production	TMS$pIFG	Word$length$(visual)	0.07515402749435976	0.07951117322625226	0.07733260036030601	2.2001212	0.025974025974025976	0.04937636620804938
**bootstrap**	semantic production	TMS$aIFG	Word$length$(visual)	0.09435792154913274	0.09872698243226723	0.09654245199069998	2.739153	0.003996003996003996	0.008524808524808525
**bootstrap**	semantic production	vertex	Word$length$(uttered)	0.16364245960691348	0.1677388744730865	0.16569066704	5.0139523	0.001998001998001998	0.004434871486894609
**bootstrap**	semantic production	TMS$pIFG	Word$length$(uttered)	0.14894439874903703	0.15312803176496298	0.151036215257	4.4752116	0.001998001998001998	0.004434871486894609
**bootstrap**	semantic production	TMS$aIFG	Word$length$(uttered)	0.11410767336875993	0.11808614153724006	0.116096907453	3.6173525	0.001998001998001998	0.004434871486894609
**bootstrap**	semantic production	vertex	Word$length$(sum)	0.1795829196387453	0.1836289299332547	0.181605924786	5.5640254	0.001998001998001998	0.004434871486894609
**bootstrap**	semantic production	TMS$pIFG	Word$length$(sum)	0.1416967471086187	0.1460732913273813	0.143885019218	4.075401	0.001998001998001998	0.004434871486894609
**bootstrap**	semantic production	TMS$aIFG	Word$length$(sum)	0.1407643809068969	0.14482116988310306	0.142792775395	4.3632483	0.001998001998001998	0.004434871486894609
**bootstrap**	picture naming w/ interf	vertex	wac$PPMI	0.2553085340648052	0.2604960732951948	0.25790230368	6.1628265	0.001998001998001998	0.004434871486894609
**bootstrap**	picture naming w/ interf	TMS$pMTG	wac$PPMI	0.29090685901491914	0.29601438866508084	0.29346062384	7.1223774	0.001998001998001998	0.004434871486894609
**bootstrap**	picture naming w/ interf	vertex	GPT2$surprisal	0.27100700955869467	0.27636658838930533	0.273686798974	6.3300815	0.001998001998001998	0.004434871486894609
**bootstrap**	picture naming w/ interf	TMS$pMTG	GPT2$surprisal	0.30454431234869067	0.3097373598313093	0.30714083609	7.3316426	0.001998001998001998	0.004434871486894609
**bootstrap**	picture naming w/ interf	vertex	Word$frequency$(neg.$visual)	0.07286970030123491	0.0782173930734851	0.07554354668736	1.7511243	0.08391608391608392	0.1484966646256969
**bootstrap**	picture naming w/ interf	TMS$pMTG	Word$frequency$(neg.$visual)	0.05442983308109419	0.0598106737283658	0.057120253404729995	1.3159096	0.16383616383616384	0.26545606292441737

Figure C17

Full stats against null hypothesis #3

**bootstrap**	picture naming w/ interf	vertex	Word$frequency$(neg.$uttered)	0.05576301469360768	0.06102835521598233	0.058395684954795	1.3748026	0.15984015984015984	0.26118562288775055
**bootstrap**	picture naming w/ interf	TMS$pMTG	Word$frequency$(neg.$uttered)	0.06379714839592542	0.06915061033275459	0.06647387936434	1.5392259	0.11388611388611389	0.19523333809048093
**bootstrap**	picture naming w/ interf	vertex	Word$frequency$(neg. sum)	0.04319742100426359	0.04867099775114442	0.045934209377704004	1.0402821	0.27972027972027974	0.41958041958041964
**bootstrap**	picture naming w/ interf	TMS$pMTG	Word$frequency$(neg. sum)	0.030434046128468197	0.03591524521029982	0.03317464566938401	0.7502687	0.43756243756243757	0.5874964196642519
**bootstrap**	picture naming w/ interf	vertex	Word$length$(visual)	0.043138267658765606	0.04835170417160038	0.045744985915182994	1.087691	0.26573426573426573	0.4033278973990437
**bootstrap**	picture naming w/ interf	TMS$pMTG	Word$length$(visual)	0.01346541522356407	0.018722679007886527	0.016094047115725298	0.37948236	0.7092907092907093	0.825356461720098
**bootstrap**	picture naming w/ interf	vertex	Word$length$(uttered)	0.03738051985976713	0.04263493762141288	0.040007728740590005	0.94385517	0.34965034965034963	0.5009915457676651
**bootstrap**	picture naming w/ interf	TMS$pMTG	Word$length$(uttered)	0.033814875226985036	0.03903955082791097	0.036427213027448	0.86427647	0.42157842157842157	0.5720357381134766
**bootstrap**	picture naming w/ interf	vertex	Word$length$(sum)	0.05615529324459866	0.061480285718825335	0.058817789481712	1.369228	0.1838161838161838	0.2953364627004794
**bootstrap**	picture naming w/ interf	TMS$pMTG	Word$length$(sum)	0.03576725865448574	0.04114575940286025	0.038456509028672994	0.8863284	0.36163836163836166	0.5124322172292652
**bootstrap**	semantic relatedness judg.	vertex	wac$PPMI	0.21131207682388886	0.21688223131611112	0.21409715407	4.764636	0.001998001998001998	0.004434871486894609
**bootstrap**	semantic relatedness judg.	TMS$rCereb	wac$PPMI	0.15711172416598526	0.1625306607870907	0.15982119247653798	3.6560016	0.001998001998001998	0.004434871486894609
**bootstrap**	semantic relatedness judg.	vertex	GPT2$surprisal	0.23634362886023705	0.2415609093277629	0.23895226909399997	5.6774464	0.001998001998001998	0.004434871486894609
**bootstrap**	semantic relatedness judg.	TMS$rCereb	GPT2$surprisal	0.20992789764397318	0.21491879439402684	0.212423346019	5.2760596	0.001998001998001998	0.004434871486894609
**bootstrap**	semantic relatedness judg.	vertex	Word$frequency$(neg.$first w.)	-0.058353923463306916	-0.05337106004287908	-0.055862491753093	-1.3897202	0.1918081918081918	0.30561969151180773
**bootstrap**	semantic relatedness judg.	TMS$rCereb	Word$frequency$(neg.$first w.)	-0.054514956620245456	-0.04943568724101455	-0.05197532193063	-1.2684752	0.2137862137862138	0.3364504348110906
**bootstrap**	semantic relatedness judg.	vertex	Word$frequency$(neg.$second w	0.19884220590891002	0.20445418626709003	0.20164819608800003	4.454144	0.001998001998001998	0.004434871486894609
**bootstrap**	semantic relatedness judg.	TMS$rCereb	Word$frequency$(neg.$second w	0.2082504315052365	0.21342850308076353	0.21083946729300002	5.0474257	0.001998001998001998	0.004434871486894609
**bootstrap**	semantic relatedness judg.	vertex	Word$frequency$(neg. sum)	0.0719526346215247	0.0770733650512153	0.07451299983637	1.803791	0.07592407592407592	0.13497613497613498
**bootstrap**	semantic relatedness judg.	TMS$rCereb	Word$frequency$(neg. sum)	0.08132625116463392	0.0862746718459181	0.08380046150527601	2.0992582	0.03396603396603397	0.06331532545124778
**bootstrap**	semantic relatedness judg.	vertex	Word$length$(first w.)	-0.026188037264938256	-0.020952903465803745	-0.023570470365371	-0.55811864	0.5594405594405595	0.7043448354923765
**bootstrap**	semantic relatedness judg.	TMS$rCereb	Word$length$(first w.)	-0.03121341170629253	-0.026104597351427474	-0.028659004528860003	-0.6953878	0.47952047952047955	0.6284500482452701
**bootstrap**	semantic relatedness judg.	vertex	Word$length$(second w.)	0.17391811658805637	0.17874767622394364	0.176332896406	4.525972	0.001998001998001998	0.004434871486894609
**bootstrap**	semantic relatedness judg.	TMS$rCereb	Word$length$(second w.)	0.15907500100481298	0.16407125500118702	0.161573128003	4.0087657	0.001998001998001998	0.004434871486894609
**bootstrap**	semantic relatedness judg.	vertex	Word$length$(sum)	0.10492514448578369	0.1100897215822163	0.107507433034	2.5804164	0.015984015984015984	0.031638464628155345
**bootstrap**	semantic relatedness judg.	TMS$rCereb	Word$length$(sum)	0.09497829104969506	0.10007277831490492	0.09752553468229999	2.3730338	0.02197802197802198	0.04240985145507759
**bootstrap**	quantity priming	vertex	wac$PPMI	0.11577015878741309	0.12011026532658692	0.117940212057	3.3685856	0.001998001998001998	0.004434871486894609
**bootstrap**	quantity priming	TMS$rSATL	wac$PPMI	0.046128448675443245	0.05012642108110875	0.048127434878276	1.492241	0.12987012987012986	0.21872863978127133
**bootstrap**	quantity priming	TMS$rIPS	wac$PPMI	0.028385924593678346	0.03254748119226166	0.030466702892970002	0.9075189	0.36363636363636365	0.5133689839572192
**bootstrap**	quantity priming	vertex	GPT2$surprisal	0.04459470400882605	0.04862126640474594	0.046607985206785996	1.4348681	0.13586413586413587	0.2268340355296877
**bootstrap**	quantity priming	TMS$rSATL	GPT2$surprisal	0.06340568898956903	0.06776164468407098	0.06558366683682	1.8663725	0.061938061938061936	0.11166298490242152
**bootstrap**	quantity priming	TMS$rIPS	GPT2$surprisal	0.05466626643219036	0.05871351617580964	0.056689891304	1.7363276	0.07192807192807193	0.12846688195525405
**bootstrap**	quantity priming	vertex	Word$frequency$(neg.$first w.)	-0.100447546995202	-0.09635663253079799	-0.098402089763	-2.9817412	0.005994005994005994	0.012577586348078151
**bootstrap**	quantity priming	TMS$rSATL	Word$frequency$(neg.$first w.)	-0.04273020467815999	-0.03872752892332001	-0.04072886680074	-1.261357	0.1958041958041958	0.310697566895914
**bootstrap**	quantity priming	TMS$rIPS	Word$frequency$(neg.$first w.)	-1.0231980171902712e-05	0.004069020394879903	0.0020293942073539998	0.061669696	0.935064935064935	0.9891596007298487
**bootstrap**	quantity priming	vertex	Word$frequency$(neg.$second w	0.025708681174587647	0.029835556287682356	0.027772118731135	0.8342068	0.3776223776223776	0.5272981563890654
**bootstrap**	quantity priming	TMS$rSATL	Word$frequency$(neg.$second w	-0.005448068221897884	-0.001351506505942116	-0.00339978736392	-0.10287701	0.8771228771228772	0.9382038574239131
**bootstrap**	quantity priming	TMS$rIPS	Word$frequency$(neg.$second w	0.01861492692806109	0.02292987966953891	0.0207724032988	0.596756	0.5594405594405595	0.7043448354923765
**bootstrap**	quantity priming	vertex	Word$frequency$(neg. sum)	-0.07538326032283238	-0.07119344068256764	-0.07328835050270001	-2.1683316	0.03396603396603397	0.06331532545124778
**bootstrap**	quantity priming	TMS$rSATL	Word$frequency$(neg. sum)	-0.039894127590637714	-0.03573199375234227	-0.03781306067148999	-1.1261904	0.25774225774225773	0.3943148484981154
**bootstrap**	quantity priming	TMS$rIPS	Word$frequency$(neg. sum)	0.013168978641918341	0.01733180655324366	0.015250392597581	0.45412838	0.6493506493506493	0.779183433389041
**bootstrap**	quantity priming	vertex	Word$length$(first w.)	-0.10350464760585025	-0.09921421231300977	-0.10135942995943001	-2.9285245	0.003996003996003996	0.008524808524808525
**bootstrap**	quantity priming	TMS$rSATL	Word$length$(first w.)	-0.04283799214686121	-0.0384935073599188	-0.040665749753390004	-1.1603167	0.23176823176823177	0.3588669395121008
**bootstrap**	quantity priming	TMS$rIPS	Word$length$(first w.)	0.0007030998958465835	0.004867913973573416	0.00278550693471	0.08290766	0.939060939060939	0.9906576939543973
**bootstrap**	quantity priming	vertex	Word$length$(second w.)	0.10539511344222544	0.10960032460217456	0.1074977190222	3.16882	0.003996003996003996	0.008524808524808525
**bootstrap**	quantity priming	TMS$rSATL	Word$length$(second w.)	0.046520824786889306	0.05083534529127068	0.04867808503907999	1.398579	0.13986013986013987	0.2324947779493234
**bootstrap**	quantity priming	TMS$rIPS	Word$length$(second w.)	0.16623918048187492	0.1703729145881251	0.168306047535	5.047116	0.001998001998001998	0.004434871486894609
**bootstrap**	quantity priming	vertex	Word$length$(sum)	0.0816253124610521	0.08583342182614791	0.0837293671436	2.466476	0.011988011988011988	0.024228403175771594
**bootstrap**	quantity priming	TMS$rSATL	Word$length$(sum)	0.03412374193967113	0.03823545134925686	0.036179596644464	1.0907553	0.27972027972027974	0.41958041958041964
**bootstrap**	quantity priming	TMS$rIPS	Word$length$(sum)	0.16224163092227334	0.16628029410972667	0.164260962516	5.041767	0.001998001998001998	0.004434871486894609
**bootstrap**	social priming	vertex	wac$PPMI	-0.022154382003487627	-0.017922613660654375	-0.020038497832071	-0.5869882	0.5574425574425574	0.7043448354923765
**bootstrap**	social priming	TMS$rSATL	wac$PPMI	0.025669067739546525	0.03015269131028548	0.027910879524916003	0.77166784	0.43156843156843155	0.5814816762185183
**bootstrap**	social priming	TMS$rIPS	wac$PPMI	0.05068382803196562	0.05495517285304637	0.052819500442505996	1.5329068	0.12987012987012986	0.21872863978127133
**bootstrap**	social priming	vertex	GPT2$surprisal	0.018842551241801783	0.022962460687842214	0.020902505964822	0.6289219	0.5254745254745254	0.6726073926073926
**bootstrap**	social priming	TMS$rSATL	GPT2$surprisal	0.049288641100972065	0.053585927536507934	0.05143728431874	1.483781	0.13186813186813187	0.22112385431162726
**bootstrap**	social priming	TMS$rIPS	GPT2$surprisal	0.054623634759567156	0.058859918040532845	0.05674177640005	1.6603665	0.1038961038961039	0.18052535699594524
**bootstrap**	social priming	vertex	Word$frequency$(neg.$first w.)	0.0489151958581522	0.05311688077614782	0.05101603831715001	1.5051137	0.14585414585414586	0.24141375865513798
**bootstrap**	social priming	TMS$rSATL	Word$frequency$(neg.$first w.)	-0.0837179610175455	-0.07957089775365447	-0.08164442938559999	-2.4404614	0.01998001998001998	0.03914452894044731
**bootstrap**	social priming	TMS$rIPS	Word$frequency$(neg.$first w.)	-0.0533990620167555	-0.04897330187834449	-0.051186181947549994	-1.4336758	0.14985014985014986	0.24696333709209245
**bootstrap**	social priming	vertex	Word$frequency$(neg.$second w	-0.032087821229919114	-0.027655712378258156	-0.029871766804088637	-0.8354809	0.42157842157842157	0.5720357381134766
**bootstrap**	social priming	TMS$rSATL	Word$frequency$(neg.$second w	-0.033472262179193496	-0.029121923257986498	-0.03129709271859	-0.8917989	0.4015984015984016	0.5547258496898785
**bootstrap**	social priming	TMS$rIPS	Word$frequency$(neg.$second w	0.021442150073163732	0.025915319140028273	0.023678734606596002	0.65618944	0.4975024975024975	0.6410770437616076
**bootstrap**	social priming	vertex	Word$frequency$(neg. sum)	0.029715448586487316	0.034020063195616686	0.031867755891052	0.9177054	0.3436563436563437	0.49610539836103745
**bootstrap**	social priming	TMS$rSATL	Word$frequency$(neg. sum)	-0.0862364546526502	-0.08175714929674979	-0.0839968019747	-2.3245459	0.023976023976023976	0.046033966033966034
**bootstrap**	social priming	TMS$rIPS	Word$frequency$(neg. sum)	-0.0349903593906432	-0.03057116316044681	-0.032780761275545	-0.9195214	0.38961038961038963	0.5420666290231508
**bootstrap**	social priming	vertex	Word$length$(first w.)	0.05220222914366232	0.05643886200099768	0.05432054557233	1.5893859	0.11388611388611389	0.19523333809048093
**bootstrap**	social priming	TMS$rSATL	Word$length$(first w.)	-0.08510244143918554	-0.08099306084549444	-0.08304775114233999	-2.5051723	0.017982017982017984	0.0354107431030508
**bootstrap**	social priming	TMS$rIPS	Word$length$(first w.)	-0.05422886240874873	-0.04988820171945127	-0.0520585320641	-1.4866959	0.12987012987012986	0.21872863978127133
**bootstrap**	social priming	vertex	Word$length$(second w.)	0.1776494292878057	0.18187827125619432	0.179763850272	5.26947	0.001998001998001998	0.004434871486894609
**bootstrap**	social priming	TMS$rSATL	Word$length$(second w.)	0.19858926934450166	0.20284361791949834	0.200716443632	5.848385	0.001998001998001998	0.004434871486894609
**bootstrap**	social priming	TMS$rIPS	Word$length$(second w.)	0.18508984053486213	0.18941079703113792	0.18725031878300002	5.37191	0.001998001998001998	0.004434871486894609
**bootstrap**	social priming	vertex	Word$length$(sum)	0.17964238315696063	0.18371858170303934	0.18168048242999998	5.525086	0.001998001998001998	0.004434871486894609
**bootstrap**	social priming	TMS$rSATL	Word$length$(sum)	0.176562443784629	0.18073311476737103	0.178647779276	5.309796	0.001998001998001998	0.004434871486894609
**bootstrap**	social priming	TMS$rIPS	Word$length$(sum)	0.17049436033861978	0.17463056826338028	0.17256246430100003	5.171661	0.001998001998001998	0.004434871486894609

Figure C18

Full stats for comparisons among conditions #1

wac_corrected_comparisons_p-vals

approach	dataset	condition_one	condition_two	model	ci_min	ci_max	t_value	raw_p	fdr_corrected_p
residualize	action feature judg.	sham	TMS$pIPL	wac$PPMI	-0.49	-0.36	-0.42530397405247744	0.000999000999000999	0.003223667089213307
residualize	action feature judg.	sham	TMS$pIPL	GPT2$surprisal	-0.47	-0.34	-0.40514574011721627	0.000999000999000999	0.003223667089213307
residualize	action feature judg.	sham	TMS$pIPL	Word$frequency$(neg. sum)	-0.2	-0.07	-0.1366880688823786	0.000999000999000999	0.003223667089213307
residualize	action feature judg.	sham	TMS$pIPL	Word$length$(sum)	0.13	0.26	0.19678390362103132	0.000999000999000999	0.003223667089213307
residualize	sound feature judg.	sham	TMS$pIPL	wac$PPMI	0.69	0.83	0.7628344581399927	0.000999000999000999	0.003223667089213307
residualize	sound feature judg.	sham	TMS$pIPL	GPT2$surprisal	-0.95	-0.81	-0.8788828630840638	0.000999000999000999	0.003223667089213307
residualize	sound feature judg.	sham	TMS$pIPL	Word$frequency$(neg. sum)	-0.61	-0.48	-0.5447002120573357	0.000999000999000999	0.003223667089213307
residualize	sound feature judg.	sham	TMS$pIPL	Word$length$(sum)	-1.13	-0.97	-1.052204278419338	0.000999000999000999	0.003223667089213307
residualize	picture naming w/ interf	vertex	TMS$pMTG	wac$PPMI	-0.98	-0.84	-0.9096126627371884	0.000999000999000999	0.003223667089213307
residualize	picture naming w/ interf	vertex	TMS$pMTG	GPT2$surprisal	-0.85	-0.71	-0.7795261177736709	0.02197802197802198	0.04240985145507759
residualize	picture naming w/ interf	vertex	TMS$pMTG	Word$frequency$(neg.$visual)	0.27	0.4	0.3387875965792799	0.000999000999000999	0.003223667089213307
residualize	picture naming w/ interf	vertex	TMS$pMTG	Word$frequency$(neg.$uttered)	-0.29	-0.17	-0.2303980134916195	0.000999000999000999	0.003223667089213307
residualize	picture naming w/ interf	vertex	TMS$pMTG	Word$frequency$(neg. sum)	0.12	0.24	0.18075233116268422	0.000999000999000999	0.003223667089213307
residualize	picture naming w/ interf	vertex	TMS$pMTG	Word$length$(visual)	0.63	0.77	0.7038616871658667	0.000999000999000999	0.003223667089213307
residualize	picture naming w/ interf	vertex	TMS$pMTG	Word$length$(uttered)	-0.07	0.05	-0.009187882428674849	0.8561438561438561	0.9297820823244551
residualize	picture naming w/ interf	vertex	TMS$pMTG	Word$length$(sum)	0.28	0.41	0.3436346061047949	0.000999000999000999	0.003223667089213307
residualize	semantic production	vertex	TMS$pIFG	wac$PPMI	-1.55	-1.38	-1.4643706309874398	0.000999000999000999	0.003223667089213307
residualize	semantic production	vertex	TMS$aIFG	wac$PPMI	-1.51	-1.34	-1.4259521372754027	0.000999000999000999	0.003223667089213307
residualize	semantic production	vertex	TMS$pIFG	GPT2$surprisal	-0.41	-0.28	-0.3452253347070806	0.000999000999000999	0.003223667089213307
residualize	semantic production	vertex	TMS$aIFG	GPT2$surprisal	-0.19	-0.07	-0.1290639600216796	0.04595404595404595	0.08443231409738586
residualize	semantic production	vertex	TMS$pIFG	Word$frequency$(neg.$visual)	-0.38	-0.25	-0.31838054949682815	0.000999000999000999	0.003223667089213307
residualize	semantic production	vertex	TMS$aIFG	Word$frequency$(neg.$visual)	0.31	0.44	0.3755342088837919	0.000999000999000999	0.003223667089213307
residualize	semantic production	vertex	TMS$pIFG	Word$frequency$(neg.$uttered)	-0.85	-0.71	-0.7819761303877203	0.000999000999000999	0.003223667089213307
residualize	semantic production	vertex	TMS$aIFG	Word$frequency$(neg.$uttered)	-0.09	0.04	-0.025579537881967544	0.3696303696303696	0.5180221238615399
residualize	semantic production	vertex	TMS$pIFG	Word$frequency$(neg. sum)	-0.82	-0.68	-0.7521599023523816	0.000999000999000999	0.003223667089213307
residualize	semantic production	vertex	TMS$aIFG	Word$frequency$(neg. sum)	0.35	0.48	0.4130713662882039	0.000999000999000999	0.003223667089213307
residualize	semantic production	vertex	TMS$pIFG	Word$length$(visual)	0.11	0.24	0.1758088955264203	0.000999000999000999	0.003223667089213307
residualize	semantic production	vertex	TMS$aIFG	Word$length$(visual)	-0.46	-0.33	-0.3931804386247016	0.000999000999000999	0.003223667089213307
residualize	semantic production	vertex	TMS$pIFG	Word$length$(uttered)	0.25	0.37	0.311119780678324	0.000999000999000999	0.003223667089213307
residualize	semantic production	vertex	TMS$aIFG	Word$length$(uttered)	1.21	1.38	1.2984874026878384	0.000999000999000999	0.003223667089213307
residualize	semantic production	vertex	TMS$pIFG	Word$length$(sum)	0.69	0.84	0.764817248279218	0.000999000999000999	0.003223667089213307
residualize	semantic production	vertex	TMS$aIFG	Word$length$(sum)	0.65	0.79	0.7184816092715638	0.000999000999000999	0.003223667089213307
residualize	quantity priming	vertex	TMS$rSATL	wac$PPMI	1.98	2.2	2.0898405405307274	0.000999000999000999	0.003223667089213307
residualize	quantity priming	vertex	TMS$rIPS	wac$PPMI	2.06	2.28	2.1686554059422196	0.000999000999000999	0.003223667089213307
residualize	quantity priming	vertex	TMS$rSATL	GPT2$surprisal	0.21	0.34	0.27246888163510724	0.000999000999000999	0.003223667089213307
residualize	quantity priming	vertex	TMS$rIPS	GPT2$surprisal	0.02	0.14	0.07834347380697466	0.058941058941058944	0.10676116336493695
residualize	quantity priming	vertex	TMS$rSATL	Word$frequency$(neg.$first w.)	-1.69	-1.51	-1.6008240936438618	0.000999000999000999	0.003223667089213307
residualize	quantity priming	vertex	TMS$rIPS	Word$frequency$(neg.$first w.)	-2.54	-2.29	-2.413948965557334	0.000999000999000999	0.003223667089213307
residualize	quantity priming	vertex	TMS$rSATL	Word$frequency$(neg.$second w.)	1.13	1.3	1.2138828144608174	0.000999000999000999	0.003223667089213307
residualize	quantity priming	vertex	TMS$rIPS	Word$frequency$(neg.$second w.)	0.35	0.48	0.4134099794627867	0.000999000999000999	0.003223667089213307
residualize	quantity priming	vertex	TMS$rSATL	Word$frequency$(neg. sum)	-0.9	-0.76	-0.8283048720197022	0.000999000999000999	0.003223667089213307
residualize	quantity priming	vertex	TMS$rIPS	Word$frequency$(neg. sum)	-2.12	-1.9	-2.0115788797582472	0.000999000999000999	0.003223667089213307
residualize	quantity priming	vertex	TMS$rSATL	Word$length$(first w.)	-1.66	-1.47	-1.5674758097888508	0.000999000999000999	0.003223667089213307
residualize	quantity priming	vertex	TMS$rIPS	Word$length$(first w.)	-2.62	-2.37	-2.4913938474523807	0.000999000999000999	0.003223667089213307
residualize	quantity priming	vertex	TMS$rSATL	Word$length$(second w.)	1.77	1.97	1.8687080432383705	0.000999000999000999	0.003223667089213307
residualize	quantity priming	vertex	TMS$rIPS	Word$length$(second w.)	-1.38	-1.21	-1.2971397788838255	0.000999000999000999	0.003223667089213307
residualize	quantity priming	vertex	TMS$rSATL	Word$length$(sum)	1.53	1.72	1.6246935623913903	0.000999000999000999	0.003223667089213307
residualize	quantity priming	vertex	TMS$rIPS	Word$length$(sum)	-1.8	-1.61	-1.7033904132356292	0.000999000999000999	0.003223667089213307
residualize	social priming	vertex	TMS$rSATL	wac$PPMI	-1.12	-0.97	-1.0473933646208144	0.000999000999000999	0.003223667089213307
residualize	social priming	vertex	TMS$rIPS	wac$PPMI	-1.67	-1.48	-1.577621261624924	0.000999000999000999	0.003223667089213307
residualize	social priming	vertex	TMS$rSATL	GPT2$surprisal	-0.79	-0.65	-0.7219426133860828	0.000999000999000999	0.003223667089213307
residualize	social priming	vertex	TMS$rIPS	GPT2$surprisal	-0.79	-0.65	-0.7216403914880227	0.000999000999000999	0.003223667089213307
residualize	social priming	vertex	TMS$rSATL	Word$frequency$(neg.$first w.)	2.94	3.24	3.087845034523482	0.000999000999000999	0.003223667089213307
residualize	social priming	vertex	TMS$rIPS	Word$frequency$(neg.$first w.)	2.25	2.49	2.3677120789631725	0.000999000999000999	0.003223667089213307
residualize	social priming	vertex	TMS$rSATL	Word$frequency$(neg.$second w.)	0.02	0.15	0.08480305598501157	0.2007992007992008	0.3173123173123173
residualize	social priming	vertex	TMS$rIPS	Word$frequency$(neg.$second w.)	-1.25	-1.09	-1.1663628709822471	0.000999000999000999	0.003223667089213307
residualize	social priming	vertex	TMS$rSATL	Word$frequency$(neg. sum)	2.64	2.91	2.7776326006550107	0.000999000999000999	0.003223667089213307
residualize	social priming	vertex	TMS$rIPS	Word$frequency$(neg. sum)	1.43	1.61	1.5224265775232593	0.000999000999000999	0.003223667089213307
residualize	social priming	vertex	TMS$rSATL	Word$length$(first w.)	2.91	3.21	3.0593146934553723	0.000999000999000999	0.003223667089213307
residualize	social priming	vertex	TMS$rIPS	Word$length$(first w.)	2.26	2.5	2.378475467163757	0.000999000999000999	0.003223667089213307
residualize	social priming	vertex	TMS$rSATL	Word$length$(second w.)	-0.49	-0.36	-0.42770489952543816	0.000999000999000999	0.003223667089213307
residualize	social priming	vertex	TMS$rIPS	Word$length$(second w.)	0.26	0.39	0.3263933903110363	0.000999000999000999	0.003223667089213307
residualize	social priming	vertex	TMS$rSATL	Word$length$(sum)	0.13	0.25	0.19157408964895048	0.000999000999000999	0.003223667089213307
residualize	social priming	vertex	TMS$rIPS	Word$length$(sum)	0.65	0.79	0.7198333683520242	0.000999000999000999	0.003223667089213307
residualize	semantic relatedness judg.	vertex	TMS$rCereb	wac$PPMI	1.1	1.27	1.1846375793531807	0.000999000999000999	0.003223667089213307
residualize	semantic relatedness judg.	vertex	TMS$rCereb	GPT2$surprisal	0.6	0.74	0.6691293248644998	0.000999000999000999	0.003223667089213307
residualize	semantic relatedness judg.	vertex	TMS$rCereb	Word$frequency$(neg.$first w.)	-0.02	0.1	0.03830492058500293	0.41058941058941056	0.56245533825605
residualize	semantic relatedness judg.	vertex	TMS$rCereb	Word$frequency$(neg.$second w.)	0.01	0.14	0.07324343557527857	0.08791208791208792	0.15485431999193466
residualize	semantic relatedness judg.	vertex	TMS$rCereb	Word$frequency$(neg. sum)	0.03	0.16	0.09497787808425667	0.053946053946053944	0.09864421292992721
residualize	semantic relatedness judg.	vertex	TMS$rCereb	Word$length$(first w.)	0.21	0.33	0.268974664771396	0.000999000999000999	0.003223667089213307
residualize	semantic relatedness judg.	vertex	TMS$rCereb	Word$length$(second w.)	0.28	0.41	0.3480028768622444	0.000999000999000999	0.003223667089213307

Figure C19

Full stats for comparisons among conditions #2

**residualize**	semantic relatedness judg.	vertex	TMS$rCereb	Word$length$(sum)	0.37	0.5	0.43066287937064746	0.000999000999000999	0.0032236670892133077
**bootstrap**	action feature judg.	sham	TMS$pIPL	wac$PPMI	-0.42	-0.29	-0.35348737313264494	0.000999000999000999	0.0032236670892133077
**bootstrap**	action feature judg.	sham	TMS$pIPL	GPT2$surprisal	-0.48	-0.35	-0.41692345286054266	0.000999000999000999	0.0032236670892133077
**bootstrap**	action feature judg.	sham	TMS$pIPL	Word$frequency$(neg. sum)	-0.24	-0.11	-0.17371681359039087	0.000999000999000999	0.0032236670892133077
**bootstrap**	action feature judg.	sham	TMS$pIPL	Word$length$(sum)	0.15	0.28	0.21448662663693127	0.000999000999000999	0.0032236670892133077
**bootstrap**	sound feature judg.	sham	TMS$pIPL	wac$PPMI	0.58	0.71	0.6446117889223292	0.000999000999000999	0.0032236670892133077
**bootstrap**	sound feature judg.	sham	TMS$pIPL	GPT2$surprisal	-0.96	-0.81	-0.8857601589286543	0.000999000999000999	0.0032236670892133077
**bootstrap**	sound feature judg.	sham	TMS$pIPL	Word$frequency$(neg. sum)	-0.67	-0.54	-0.6029210507076723	0.000999000999000999	0.0032236670892133077
**bootstrap**	sound feature judg.	sham	TMS$pIPL	Word$length$(sum)	-1.25	-1.09	-1.1725164949817355	0.000999000999000999	0.0032236670892133077
**bootstrap**	picture naming w/ interf	vertex	TMS$pIFG	wac$PPMI	-1.74	-1.55	-1.6472164540836678	0.000999000999000999	0.0032236670892133077
**bootstrap**	picture naming w/ interf	vertex	TMS$aIFG	wac$PPMI	-1.88	-1.68	-1.7779996049580187	0.000999000999000999	0.0032236670892133077
**bootstrap**	picture naming w/ interf	vertex	TMS$pIFG	GPT2$surprisal	-0.44	-0.31	-0.37283370594610893	0.000999000999000999	0.0032236670892133077
**bootstrap**	picture naming w/ interf	vertex	TMS$aIFG	GPT2$surprisal	-0.54	-0.41	-0.47379951772386714	0.000999000999000999	0.0032236670892133077
**bootstrap**	picture naming w/ interf	vertex	TMS$pIFG	Word$frequency$(neg.$visual)	-0.5	-0.37	-0.4337242485521215	0.000999000999000999	0.0032236670892133077
**bootstrap**	picture naming w/ interf	vertex	TMS$aIFG	Word$frequency$(neg.$visual)	0.45	0.59	0.520233188043064	0.002997002997002997	0.006538915629824721
**bootstrap**	picture naming w/ interf	vertex	TMS$pIFG	Word$frequency$(neg.$uttered)	-1.02	-0.87	-0.9421635948706404	0.000999000999000999	0.0032236670892133077
**bootstrap**	picture naming w/ interf	vertex	TMS$aIFG	Word$frequency$(neg.$uttered)	-0.02	0.11	0.04425725635553081	0.34965034965034963	0.5009915457676651
**bootstrap**	semantic production	vertex	TMS$pIFG	Word$frequency$(neg. sum)	-1.31	-1.15	-1.2294799493952118	0.000999000999000999	0.0032236670892133077
**bootstrap**	semantic production	vertex	TMS$aIFG	Word$frequency$(neg. sum)	0.51	0.64	0.5743482386916938	0.01098901098901099	0.022445639466916065
**bootstrap**	semantic production	vertex	TMS$pIFG	Word$length$(visual)	0.29	0.41	0.3500516518942549	0.000999000999000999	0.0032236670892133077
**bootstrap**	semantic production	vertex	TMS$aIFG	Word$length$(visual)	-0.28	-0.15	-0.2124936368397289	0.6923076923076923	0.812985179957657
**bootstrap**	semantic production	vertex	TMS$pIFG	Word$length$(uttered)	0.37	0.5	0.4385422442310122	0.002997002997002997	0.006538915629824721
**bootstrap**	semantic production	vertex	TMS$aIFG	Word$length$(uttered)	1.43	1.61	1.5217499143185884	0.000999000999000999	0.0032236670892133077
**bootstrap**	semantic production	vertex	TMS$pIFG	Word$length$(sum)	1.03	1.19	1.1089269160716058	0.000999000999000999	0.0032236670892133077
**bootstrap**	semantic production	vertex	TMS$aIFG	Word$length$(sum)	1.11	1.27	1.186976758970205	0.000999000999000999	0.0032236670892133077
**bootstrap**	semantic production	vertex	TMS$pMTG	wac$PPMI	-0.93	-0.78	-0.8558500311199692	0.000999000999000999	0.0032236670892133077
**bootstrap**	semantic production	vertex	TMS$pMTG	GPT2$surprisal	-0.86	-0.71	-0.7854756227292347	0.000999000999000999	0.0032236670892133077
**bootstrap**	semantic production	vertex	TMS$pMTG	Word$frequency$(neg.$visual)	0.36	0.49	0.4255235907203821	0.000999000999000999	0.0032236670892133077
**bootstrap**	semantic production	vertex	TMS$pMTG	Word$frequency$(neg.$uttered)	-0.25	-0.13	-0.18850488125004508	0.01098901098901099	0.022445639466916065
**bootstrap**	semantic production	vertex	TMS$pMTG	Word$frequency$(neg. sum)	0.23	0.35	0.28862305170246716	0.000999000999000999	0.0032236670892133077
**bootstrap**	semantic production	vertex	TMS$pMTG	Word$length$(visual)	0.63	0.77	0.7017101105995593	0.000999000999000999	0.0032236670892133077
**bootstrap**	semantic production	vertex	TMS$pMTG	Word$length$(uttered)	0.02	0.15	0.08466793092697913	0.12887112887112886	0.21872863978127133
**bootstrap**	semantic production	vertex	TMS$pMTG	Word$length$(sum)	0.41	0.54	0.4713819479972672	0.000999000999000999	0.0032236670892133077
**bootstrap**	quantity priming	vertex	TMS$rCereb	wac$PPMI	1.14	1.31	1.223780215429802	0.000999000999000999	0.0032236670892133077
**bootstrap**	quantity priming	vertex	TMS$rCereb	GPT2$surprisal	0.58	0.71	0.6438185286471736	0.013986013986013986	0.028118478380258487
**bootstrap**	quantity priming	vertex	TMS$rCereb	Word$frequency$(neg.$first w.)	-0.16	-0.03	-0.09572432923530212	0.056943056943056944	0.10363096619020791
**bootstrap**	quantity priming	vertex	TMS$rCereb	Word$frequency$(neg.$second w.)	-0.27	-0.15	-0.21091134633699013	0.000999000999000999	0.0032236670892133077
**bootstrap**	quantity priming	vertex	TMS$rCereb	Word$frequency$(neg. sum)	-0.29	-0.17	-0.22852799490509543	0.000999000999000999	0.0032236670892133077
**bootstrap**	quantity priming	vertex	TMS$rCereb	Word$length$(first w.)	0.06	0.18	0.12189132326556665	0.007992007992007992	0.01658881658881659
**bootstrap**	quantity priming	vertex	TMS$rCereb	Word$length$(second w.)	0.31	0.44	0.37217511155211347	0.000999000999000999	0.0032236670892133077
**bootstrap**	quantity priming	vertex	TMS$rCereb	Word$length$(sum)	0.18	0.3	0.24109825225349366	0.02097902097902098	0.04089311703524901
**bootstrap**	quantity priming	vertex	TMS$rSATL	wac$PPMI	1.96	2.18	2.0730148920979534	0.000999000999000999	0.0032236670892133077
**bootstrap**	quantity priming	vertex	TMS$rIPS	wac$PPMI	2.42	2.68	2.549034403037493	0.000999000999000999	0.0032236670892133077
**bootstrap**	quantity priming	vertex	TMS$rSATL	GPT2$surprisal	-0.63	-0.49	-0.5605142427688048	0.000999000999000999	0.0032236670892133077
**bootstrap**	quantity priming	vertex	TMS$rIPS	GPT2$surprisal	-0.37	-0.25	-0.30942927961626177	0.000999000999000999	0.0032236670892133077
**bootstrap**	quantity priming	vertex	TMS$rSATL	Word$frequency$(neg.$first w.)	-1.86	-1.67	-1.765655592297993	0.000999000999000999	0.0032236670892133077
**bootstrap**	quantity priming	vertex	TMS$rIPS	Word$frequency$(neg.$first w.)	-3.19	-2.9	-3.0460520065611307	0.000999000999000999	0.0032236670892133077
**bootstrap**	quantity priming	vertex	TMS$rSATL	Word$frequency$(neg.$second w.)	0.86	1.01	0.9393033054431862	0.000999000999000999	0.0032236670892133077
**bootstrap**	quantity priming	vertex	TMS$rIPS	Word$frequency$(neg.$second w.)	0.14	0.27	0.20541632025394987	0.000999000999000999	0.0032236670892133077
**bootstrap**	social priming	vertex	TMS$rSATL	Word$frequency$(neg. sum)	-1.13	-0.98	-1.052529615576613	0.000999000999000999	0.0032236670892133077
**bootstrap**	social priming	vertex	TMS$rIPS	Word$frequency$(neg. sum)	-2.76	-2.5	-2.6266712613850065	0.000999000999000999	0.0032236670892133077
**bootstrap**	social priming	vertex	TMS$rSATL	Word$length$(first w.)	-1.84	-1.64	-1.741708362129087	0.000999000999000999	0.0032236670892133077
**bootstrap**	social priming	vertex	TMS$rIPS	Word$length$(first w.)	-3.2	-2.9	-3.051846147794543	0.000999000999000999	0.0032236670892133077
**bootstrap**	social priming	vertex	TMS$rSATL	Word$length$(second w.)	1.61	1.81	1.7106433418889968	0.000999000999000999	0.0032236670892133077
**bootstrap**	social priming	vertex	TMS$rIPS	Word$length$(second w.)	-1.91	-1.71	-1.8069028141181096	0.000999000999000999	0.0032236670892133077
**bootstrap**	social priming	vertex	TMS$rSATL	Word$length$(sum)	1.33	1.5	1.4161335347128974	0.000999000999000999	0.0032236670892133077
**bootstrap**	social priming	vertex	TMS$rIPS	Word$length$(sum)	-2.54	-2.3	-2.419298308334551	0.000999000999000999	0.0032236670892133077
**bootstrap**	social priming	vertex	TMS$rSATL	wac$PPMI	-1.45	-1.28	-1.3627418842993826	0.000999000999000999	0.0032236670892133077
**bootstrap**	social priming	vertex	TMS$rIPS	wac$PPMI	-2.24	-2.01	-2.1232121378161355	0.000999000999000999	0.0032236670892133077
**bootstrap**	social priming	vertex	TMS$rSATL	GPT2$surprisal	-0.97	-0.83	-0.8987310395635648	0.000999000999000999	0.0032236670892133077
**bootstrap**	social priming	vertex	TMS$rIPS	GPT2$surprisal	-1.14	-0.99	-1.06269195262092	0.000999000999000999	0.0032236670892133077
**bootstrap**	social priming	vertex	TMS$rSATL	Word$frequency$(neg.$first w.)	3.75	4.12	3.9374011143837255	0.000999000999000999	0.0032236670892133077
**bootstrap**	social priming	vertex	TMS$rIPS	Word$frequency$(neg.$first w.)	2.79	3.08	2.934475477107718	0.000999000999000999	0.0032236670892133077
**bootstrap**	social priming	vertex	TMS$rSATL	Word$frequency$(neg.$second w.)	-0.02	0.1	0.040214118288537666	0.26873126873126874	0.4062708944598708
**bootstrap**	social priming	vertex	TMS$rIPS	Word$frequency$(neg.$second w.)	-1.58	-1.4	-1.490082342758317	0.000999000999000999	0.0032236670892133077
**bootstrap**	semantic relatedness judg.	vertex	TMS$rSATL	Word$frequency$(neg. sum)	3.11	3.42	3.2679482264066837	0.000999000999000999	0.0032236670892133077
**bootstrap**	semantic relatedness judg.	vertex	TMS$rIPS	Word$frequency$(neg. sum)	1.73	1.94	1.8361733405752767	0.000999000999000999	0.0032236670892133077
**bootstrap**	semantic relatedness judg.	vertex	TMS$rSATL	Word$length$(first w.)	3.89	4.27	4.078080183387404	0.000999000999000999	0.0032236670892133077
**bootstrap**	semantic relatedness judg.	vertex	TMS$rIPS	Word$length$(first w.)	2.92	3.22	3.073072604224002	0.000999000999000999	0.0032236670892133077
**bootstrap**	semantic relatedness judg.	vertex	TMS$rSATL	Word$length$(second w.)	-0.68	-0.54	-0.6120338026124811	0.002997002997002997	0.006538915629824721
**bootstrap**	semantic relatedness judg.	vertex	TMS$rIPS	Word$length$(second w.)	-0.28	-0.15	-0.21696751345164028	0.5444555444555444	0.6922878446057253
**bootstrap**	semantic relatedness judg.	vertex	TMS$rSATL	Word$length$(sum)	0.03	0.15	0.091119450665283	0.21978021978021978	0.34168260888908664
**bootstrap**	semantic relatedness judg.	vertex	TMS$rIPS	Word$length$(sum)	0.21	0.34	0.2751168735778509	0.03596403596403597	0.06671589280284933
